# Functional Characterization of Bacteria Isolated from Ancient Arctic Soil Exposes Diverse Resistance Mechanisms to Modern Antibiotics

**DOI:** 10.1371/journal.pone.0069533

**Published:** 2015-03-25

**Authors:** Gabriel G. Perron, Lyle Whyte, Peter J. Turnbaugh, Jacqueline Goordial, William P. Hanage, Gautam Dantas, Michael M. Desai

**Affiliations:** 1 FAS Center for Systems Biology, Harvard University, 52 Oxford Street, Cambridge, Massachusetts, 02138, United States of America; 2 Department of Evolutionary and Organismic Biology, Harvard University, 52 Oxford Street, Cambridge, Massachusetts, 02138, United States of America; 3 Biology Program, Bard College, 30 Campus Road, Annandale-on-Hudson, New York, 12504, United States of America; 4 Department of Natural Resource Sciences, McGill University, Macdonald Campus, 21,111 Lakeshore, Ste-Anne-de-Bellevue, Quebec, H9X 3V9, Canada; 5 Department of Microbiology and Immunology, Hooper Foundation, University of California San Francisco, 513 Parnassus Ave, San Francisco, California, 94143, United States of America; 6 Department of Epidemiology, Harvard School of Public School, 677 Huntington Avenue, Boston, Massachusetts, 02115, United States of America; 7 Center for Genome Sciences and Systems Biology, Washington University School of Medicine, 4444 Forest Park Avenue, St. Louis, Missouri, 63108, United States of America; 8 Department of Pathology and Immunology, Washington University School of Medicine, 4444 Park Forest Avenue, St. Louis, Missouri, 63108, United States of America; 9 Department of Physics, Harvard University, Cambridge, Massachusetts, 02138, United States of America; University of Edinburgh, UNITED KINGDOM

## Abstract

Using functional metagenomics to study the resistomes of bacterial communities isolated from different layers of the Canadian high Arctic permafrost, we show that microbial communities harbored diverse resistance mechanisms at least 5,000 years ago. Among bacteria sampled from the ancient layers of a permafrost core, we isolated eight genes conferring clinical levels of resistance against aminoglycoside, β-lactam and tetracycline antibiotics that are naturally produced by microorganisms. Among these resistance genes, four also conferred resistance against amikacin, a modern semi-synthetic antibiotic that does not naturally occur in microorganisms. In bacteria sampled from the overlaying active layer, we isolated ten different genes conferring resistance to all six antibiotics tested in this study, including aminoglycoside, β-lactam and tetracycline variants that are naturally produced by microorganisms as well as semi-synthetic variants produced in the laboratory. On average, we found that resistance genes found in permafrost bacteria conferred lower levels of resistance against clinically relevant antibiotics than resistance genes sampled from the active layer. Our results demonstrate that antibiotic resistance genes were functionally diverse prior to the anthropogenic use of antibiotics, contributing to the evolution of natural reservoirs of resistance genes.

## Introduction

The evolution and spread of antibiotic resistance in pathogenic bacteria is one of the most urgent challenges in public health today [1,2]. Although resistance genes are now widespread in most microbial communities [3–7], whether the extensive diversity of resistance in environmental reservoirs and pathogenic bacteria is the result of human activity is controversial [8,9]. Rare studies considering multiple clinical isolates that pre-date the anthropogenic use of antibiotics show that resistance was uncommon in pathogenic bacteria such as *Salmonella*, *Klebsiella*, and *Escherichia* [10,11]. Furthermore, the novel evolution of antibiotic resistance through genetic mutations is well documented in clinical [12,13] and laboratory [14,15] populations of bacteria [16]. Similarly, microbial communities found in human-impacted environments such as water streams surrounding hospitals tend to show high levels of resistance genes [17,18]. Thus, the impact of antibiotic pollution on microbial communities is undeniable [19].

However, most antibiotics used in medicine today are derived from biomolecules and secondary metabolites produced by soil-dwelling microorganisms [20]. While the biosynthesis and the role of antibiotics in microbial ecosystems are a matter of active investigation [21,22], even small concentrations of antibiotic substance can lead to the evolution of high-level resistance in laboratory environments [23]. Therefore, many specialized resistance genes likely evolved in response to the natural production of antibiotics in microorganisms [24]. Indeed, genomic and phylogenetic analyses of β-lactamases, a group of enzymes that degrade penicillin and other β-lactam antibiotics, predict that precursors of the enzymes originated and diversified in bacteria millions of years ago [25,26]. The presence of antibiotic-resistant phenotypes in populations of bacteria isolated from human activity strongly supports this hypothesis [6,27].

In attempt to directly study bacteria from the pre-antibiotic era, there has been an increased interest in studying microbial communities found in ancient glaciers and permafrost [28]. Under thick layers of ice and soil, bacteria found in permafrost have been unaffected by physical and biological factors experienced at the surface for thousands of years [29]. Given current coring and sampling methods, it is now possible to extract from such ancient milieus culturable cells or DNA free from surface contaminants [30–32]. Using an extensive series of PCR-based analyses, D’Costa and colleagues conducted a metagenomic survey of ancient Alaskan soil looking for the presence of resistance genes’ molecular signatures [33]. The authors found multiple DNA fragments with similarity to genes associated with resistance against tetracyclines, β-lactams and vancomycin in modern bacteria, confirming that genes homologous to resistance genes existed in ancient bacteria [33].

DNA fragments, however, cannot confirm functional resistance against antibiotics, let alone whether they protect against the clinical use of antibiotics, for two main reasons [34]. First, the presence of DNA fragments similar to the sequence of known resistance genes is not sufficient to ensure the functional expression of a resistance phenotype [35]. Second, the sequences of many resistance genes show high levels of similarity to genes that carry out other cellular functions [19]. For example, bacterial efflux pumps of the resistance-nodulation-division (RND) superfamily can confer resistance to antibiotics, transport hydrophobic proteins involved in cell division and nodulation, or both [36]. Likewise, activation of certain RND-type efflux pumps in Gram-negative bacteria can confer sub-clinical resistance against quinolones, a synthetic group of antibiotics, under stress conditions [37]. Furthermore, analyses based on DNA sequence identity solely are limited to known resistance genes [3], ignoring the wide array of other resistance mechanisms that could have existed in the past. Although unspecified resistance genes from ancient bacteria are less likely to be of immediate concern in medical practice, they may have provided the necessary advantage for specific bacterial lineages to survive up to this day, contributing to the development of resistance reservoirs [6].

**Fig 1:**
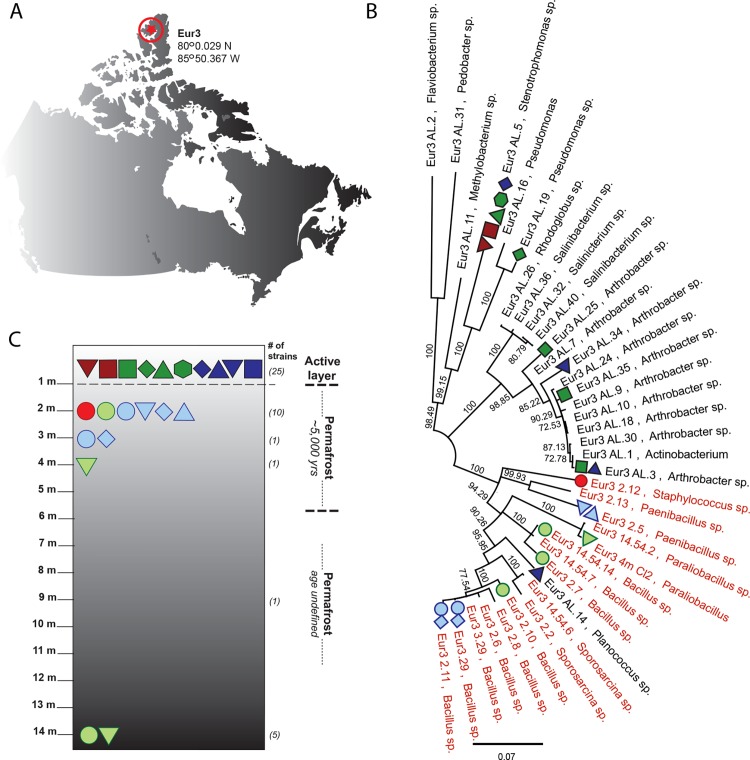
Distribution of antibiotic resistance genes isolated from ancient permafrost bacteria and its overlaying active layer. A) Samples were collected from Eureka on Ellesmere Island, Canada. B) Antibiotic resistance genes isolated using functional metagenomics were traced back to bacterial strains isolated at different depths of a single 14-m core. C) Resistance genes were mapped onto the 16S rRNA gene phylogeny of all ancient (red) and modern (black) bacterial isolates used in this study. Each resistance gene is represented by a unique color and shape combination: resistance to β-lactams (red), tetracyclines (green), and aminoglycosides (blue) as explained in **Fig. 2**. We could not identify the host strain for two resistance genes, most likely because we could not revive three ancient strains.

The functional characterization of resistance genes found in ancient permafrost environments would provide a unique window on the origins and evolution of antibiotic resistance in bacteria [8,9]. A previous study of ancient Siberian soils reported the presence of resistance to aminoglycoside, chloramphenicol and tetracyclines among Gram-positive and Gram-negative bacteria [38], but the results were met with serious criticisms [33]. Here, we use functional metagenomics to revive resistance genes associated with culturable bacteria collected from a single 14-m deep core of the Canadian high Arctic permafrost (Fig. 1A). Even though limited in scope, the study of culturable bacteria from a single permafrost core provides information on the diversity and possible sources of antibiotic resistance genes prior to the introduction of antibiotics in modern medicine.

## Materials and Methods

### Isolation of Bacteria Strains

A single core of the Canadian high Arctic was collected in May 2003 at Eureka (80°0.029 N, 85°50.367 W85°50.367 W), Ellesmere Island, Nunavut, Canada (Fig. 1A) [39]. The field research was done with the permission of the Government of Nunavut through a Territorial Scientific Research Licence to Prof. Whyte (McGill University) as issued on an annual basis by the Nunavut Research Institute from 2003 to 2012. Core Eur3 covers a depth of 14 meters and includes the active layer of the Arctic soil as well as permafrost (Fig. 1B). The geology of the core is related to sediments from the late Meistocene and subsequent Holocene and was estimated to be between 5,000 and 6,000 years old [39]. Permafrost is ground that remains at or below 0°C for at least two years [28]. During summer months, air temperatures rise above 0°C producing thaw of a thin layer at the ground surface, called the active layer. The boundary between the active layer and permafrost is the permafrost table. The permafrost table acts as a physical and biogeochemical barrier that limits infiltration of both surface water and external environmental factors [29]. At the Ellesmere Island site where the permafrost samples were obtained, the active layer reaches a depth of ∼ 50–60 cm (which has been and is readily measured with a permafrost probe) and where the underlying permafrost has an ambient and very stable temperature of −16°C [39]. Hence, there would be extremely little if any migration of microorganisms from the active layer through the permafrost table into the underlying permafrost over the last 5000 years. On the other hand there would be movement of bacteria throughout the active layer when it is thawed.

Material was collected from each 1-m subsection of the core using the sterility controls and authenticity methods described previously [30]. Culturable heterotrophic bacteria were isolated from 5 g of each subcore using the serial plate dilution as described in [39]. Plates were incubated at 37, 25 and 5°C until growth of new colonies was no longer detected. For each strain, we sequenced the first 800 base pairs of the16S rRNA gene amplified using primers 8F and 1492R (Table A in S1 File). The phylogenic profile of each strain was determined using SeqMatch from the Ribosomal Database Project release 10 [40]. The phylogenetic profiling of five strains was confirmed in two independent laboratories. The GenBank accession numbers of the 16S rRNA sequences are presented in Table D and Table E in S1 File when available.

To validate previous growth observations, two bacterial strains, Eur3 2.8 and Eur3 2.12, were grown on Tryptic Soy Agar (DB) and incubated for one month at 5, 21, and 37°C. We also tested for possible contamination of strain Eur3 2.12 by comparing the strain’s growth to that of two possible *Staphylococcus* contaminants: *S*. *aureus* and *S*. *epidermidis*. We used *S*. *aureus* strain MCD01 and *S*. *epidermidis* strain MCD02 (Table A in S1 File) and recorded growth at 5°C every two weeks using optical density (OD_600_). Finally, we confirmed the phylogenetic profiling of strain Eur3 2.12 at different time points over the course of the experiment using 16S rRNA gene sequencing using primers 8F and 1492R.

### Construction of Metagenomic Libraries

All bacterial strains were grown in 200 μL of Tryptic Soy Broth for 72 hours at room temperature before cells were harvested by centrifugation and DNA was extracted using the UltraClean Microbial DNA Isolation Kit (MoBio Laboratories, Carlsbad, CA USA). We then combined in equal proportion the DNA of all strains derived from the active layer into a single pool (hereafter referred to as “AL” for active layer) and all strains derived from the permafrost into another pool (hereafter referred to as “P” for permafrost). We constructed one metagenomics library for each pool according to the protocols described in [3]. Briefly, 10 μg of DNA were sheared using the automated Covaris S220 DNA shearer instrument using miniTUBE clear (Covaris, Woburn, MA USA) tubes and protocol for shearing of 1.5–2.5 kp fragments. Sheared DNA was end-repaired using the End-It repair kit (Epicentre, Madison, WI USA) and size-selected (1200–4000 bp) by electrophoresis through a 1% low melting point agarose gel in 1.0 X TE buffer. Size-selected and end-repaired DNA fragments were then ligated into the linearized pZE21 MCS 1 vector (Table A in S1 File) at the HincII site using the Fast Link Ligation kit (Epicentre). The ligation product was purified and resuspended in sterile deionized water before being transformed by electroporation into 20 μL of *E*. *coli* MegaX DH10B cells (Invitrogen, Grand Island, NY USA). Transformed cells were recovered in 1 mL of SOC medium (Invitrogen) and incubated with vigorous shaking for one hours at 37°C. Libraries were diluted by plating out 1 μL, 0.1 and 0.01 μL of recovered cells onto LB agar plates containing 50 μg/mL kanamycin. For each library, insert size distribution was estimated by gel electrophoresis of PCR products obtained by amplifying the insert of twelve colonies using primers flanking the multiple cloning site of the pZE21 MCS1 vector (Table A in S1 File). The total size of each library was determined by multiplying the average PCR based insert size with the number of colony forming units (CFU) in a given library and varied between 2–5 X 10^9^ base pairs. The rest of the recovered cells were inoculated into 9 mL of LB containing 50 μg/mL kanamycin and amplified over night at 37°C. The overnight cultures were frozen with 20% glycerol and kept at −80°C before subsequent analyses. We constructed a negative control library using the genomic DNA of the antibiotic-sensitive *E*. *coli* strain MegaX DH10B (Invitrogen) to screen for the possibility of exogenous antibiotic resistance gene contamination during genome extraction or library constructions [41,42]. No resistance genes were detected in the negative control library at any time during this study.

### Screening for Antibiotic Resistance

For each library, we plated 100 μL of library freezer stock, corresponding to 1.0 x 10^7^ CFU, onto LB agar plates containing binary combinations of kanamycin (50 μg/mL) and one of the six antibiotics described in Table 1. Each antibiotic was supplemented to the media at the minimal concentration required to completely inhibit the growth of the control library (Table 1). We incubated the plates at 37°C and recorded growth after 24 hours. Depending on library size, each unique clone in the libraries screened was plated out in 10–100 copies, ensuring that every clone containing an insert conferring resistance was likely to be sampled. When growth was observed, five colonies were randomly picked and streaked onto LB plates containing kanamycin and the same antibiotic on which they were selected. We confirmed that the inserts indeed conferred resistance by extracting and transforming each plasmid construct into new cultures of *E*. *coli* MegaX DH10B cells (Invitrogen). For each resistance clone, a single colony was picked and stored at −80°C in 20% glycerol for later analyses.

**Table 1:** List of antibiotics and minimal inhibitory concentration (MIC) used in this study.

Class	Antibiotic	Origin	MIC (μg/mL)	
			LB + agar	LB
Aminoglycoside	Sisomicin (SIS) [43]	*Micromonospora inyoensis*	4	1
	Amikacin (AMK) [44]	Semi-synthetic	100	32
Beta-lactamase	Penicillin (PEN) [45]	*Penicillium notatum*	50	32
	Carbenicillin (CAR) [46], [47]	Semi-synthetic	80	16
Tetracycline	Tetracycline (TET) [48]	*Streptomyces aureofaciens*	8	1
	Doxycycline (DOX) [49]	Semi-synthetic	4	0.5

Origin indicates whether the antibiotic is produced naturally by a microorganism or is modified in the laboratory.

### Measuring Resistance and Cross-Resistance

To measure the level of resistance and cross-resistance, we determined the minimal inhibitory concentration (MIC) of each clone against all six antibiotics. For each antibiotic, MIC is defined as the lowest concentration of that antibiotic that inhibits at least 90% of the bacteria’s normal growth and was estimated from the mode of four sets of replicates challenged with a dilution of twelve antibiotic concentrations: 0.0; 0.5, 1.0; 2.0; 4.0; 8.0; 16.0; 32.0; 64.0; 128.0; 256.0; 512.0; and 1024.0 μg/mL. After incubation at 37°C for 20 hours, we measured growth as optical density (OD_600_) using the SpectraMax Plus 384 absorbance plate reader (Molecular Devices, Sunnvale, CA USA). As recommended by guideline protocols for microbroth dilution, all clones were grown to a similar density before being inoculated to the twelve antibiotic dilutions to control for initial population size (approx. 1000 cells) and variation in growth phase [50].

### Sequencing Resistance Genes

**Fig 2:**
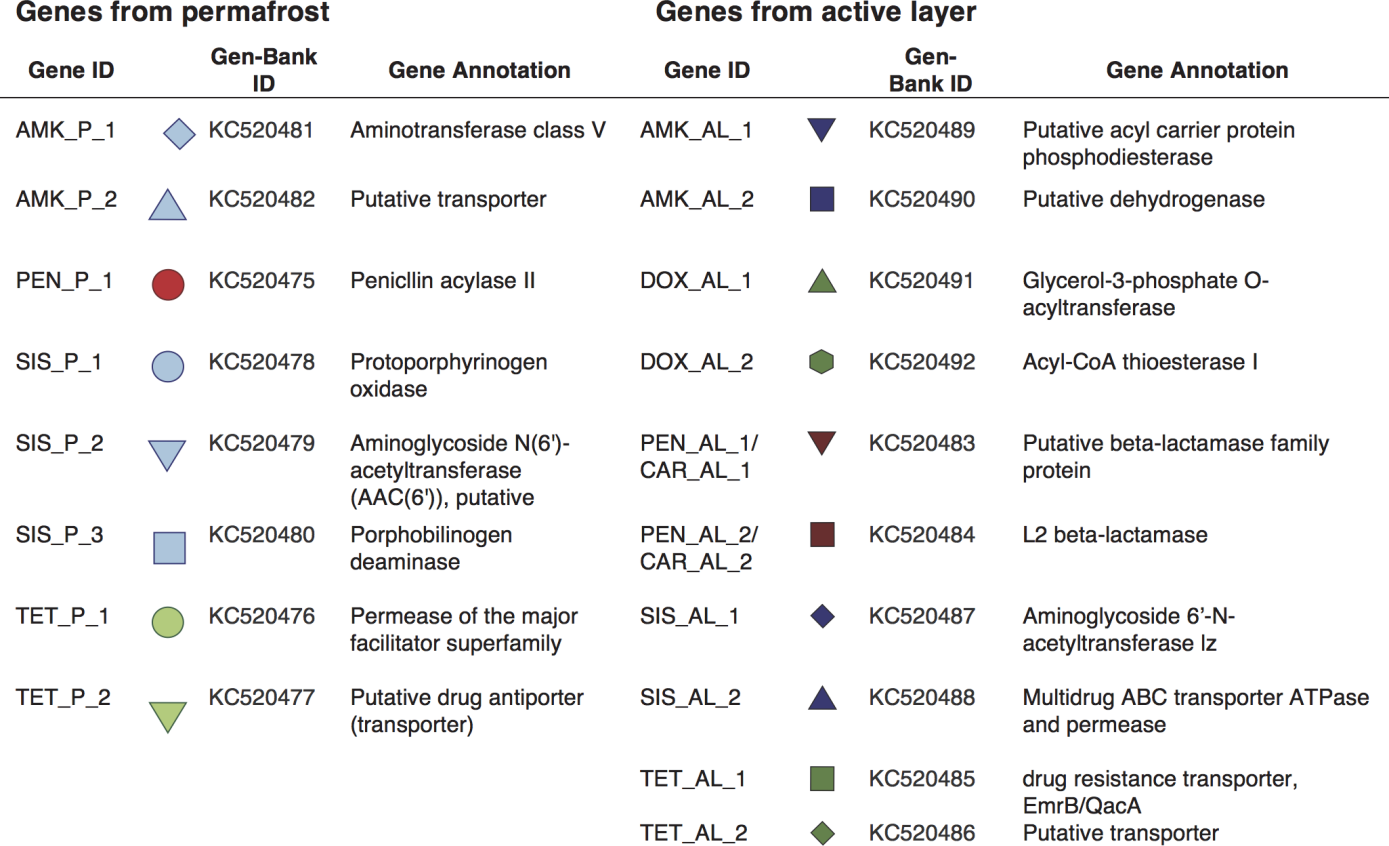
List of resistance genes and their predicted annotation.

All selected resistance clones were sequenced bi-directionally using primers flanking the *Hinc*II region of the extracted plasmid (Table A in S1 File). Sanger sequencing was performed by Genewiz (Cambridge, MA USA). Sequence corresponding to the cloning vector was removed using Geneious 5.5.4 (http://www.geneious.com). The GenBank accession number for each resistance gene can be found in Fig. 2. To estimate our sequencing error rate, the plasmids of five unique clones were sent for re-sequencing; error rate was estimated to be much lower than 0.5% over 5000 base pairs, meaning that sequencing error are negligible in our study.

### Identifying Host Strains

We designed specific primers for every resistance insert (Table B & C in S1 File) and conducted diagnosis PCRs on the extracted DNA of every bacteria strain used in this study. We used the original amplicon of the insert conferring resistance as a positive control and the DNA of *E*. *coli* MegaX DH10B as a negative control. The presence of positive PCR products was verified through low melting agarose (1%) gel electrophoresis and sequencing. Confirmation PCR was considered positive when the sequences of the amplicon and the positive control were more than 99% identical at the nucleotide level. Primers were designed using MPprimers [51] and their specificity was tested using the MFEprimer software and the *E*. *coli* genomic DNA database [52].

### Bioinformatics

For each insert, open reading frames were identified and annoted using Glimmer 3.0, and compared to the GenBank non-redundant (nr) nucleotide database (February 20, 2012) using tblastx. For each query, the GenBank ID and the alignment coordinates for the top scoring hit as well as the top scoring hit derived from a clinical pathogenic isolate was obtained. Global sequence alignment and corresponding percentage identities between the query and the obtained sequences were computed using the clustalW algorithm at the nucleotide level and the amino acid level within the annotated frame. When multiple annotate features were obtained for a query sequence, only the sequence most similar to the query at the nucleotide level was retained (Table G and H in S1 File). We then constructed a phylogeny from the multiple alignment (clustalW) of every fourth sequence of the top 100 hits (for a total of 25 unique sequences). Genetic distances were estimate using the Juke-Cantor algorithm and an unrooted consensus tree (70% of 10,000 bootstraps) was constructed using the neighbor-joining algorithm. All analyses were computed in Geneious 5.5.4 [53].

### Environmental Metagenomics

We downloaded the complete metagenomic sequences of predicted genes of eight environmental metagenomes available on MG-RAST (http://metagenomics.anl.gov/), including metagenomic survey of samples isolated from the 2-m subsection and the active layer of the same Eur3 core used in this study and twenty human intestinal gut microbiota (Table I in S1 File). The metagenomes were mounted as individual database in the Geneious 5.5.4 software to which the amino acid sequence of each resistant insert was compared using blastp. The number of significant hits (as defined by an *E*-value cutoff equal or inferior to 10^−5^) was recorded for each database. We also used ResFinder, a newly developed web-based method that uses BLAST to identify acquired antimicrobial resistance genes from a custom database [54]. We used the replicated DNA sequence file available on the MG-RAST server for both the 2-m subsection and the active layer (Table I in S1 File).

## Results

### Isolating antibiotic resistance genes

In a previous study [39], nineteen bacteria strains were isolated from subsections of a permafrost core that is estimated to be 5000–6000 years old, and twenty-one bacteria strains were sampled from the overlaying active layer soil at the surface of the permafrost (Fig. 1B). Using a fragment of the 16S rRNA gene, we typed bacterial isolates to the genus level when possible. We thus identified seven genera among bacteria isolated from ancient permafrost (Table D in S1 File), including many isolates belonging to the Bacilli, a class of ubiquitous Gram-positive Firmicute that includes free-living as well as pathogenic species. We identified eleven different genera among active layer bacteria (Table E in S1 File), including isolates of *Arthrobacter*, a common soil Gram-positive Actinobacteria, and *Stenotrophomonas*, a Gram-negative bacterium that includes soil and pathogenic species. Despite differences in phylogenetic composition (Fig. 1C), the two communities are representative of culturable microbial communities normally associated with permafrost environments [55].

We then screened for antibiotic resistance genes in two metagenomic libraries constructed from the pooled DNA of the permafrost strains (Table D in S1 File) and another constructed from the DNA of the active layer bacteria (Table E in S1 File). Antibiotic-resistant clones were selected by plating 1–7 x 10^7^ unique constructs from each library on Luria broth agar containing one of six antibiotics belonging to one of the three following antimicrobial classes: aminoglycosides, β-lactams and tetracyclines (Table 1). For each antibiotic class, we selected one molecule produced by microorganisms (referred to as native antibiotics), and another derived from synthetic modifications of a native antibiotic (referred to as semi-synthetic antibiotics). Positive inserts from resistant clones were functionally characterized, sequenced and annotated. In total, we found twenty unique inserts among the fifty inserts that we sequenced (Table F in S1 File). As we sequenced the inserts, the number of novel resistance genes quickly saturated within both libraries (Fig. A in S1 File).

**Fig 3:**
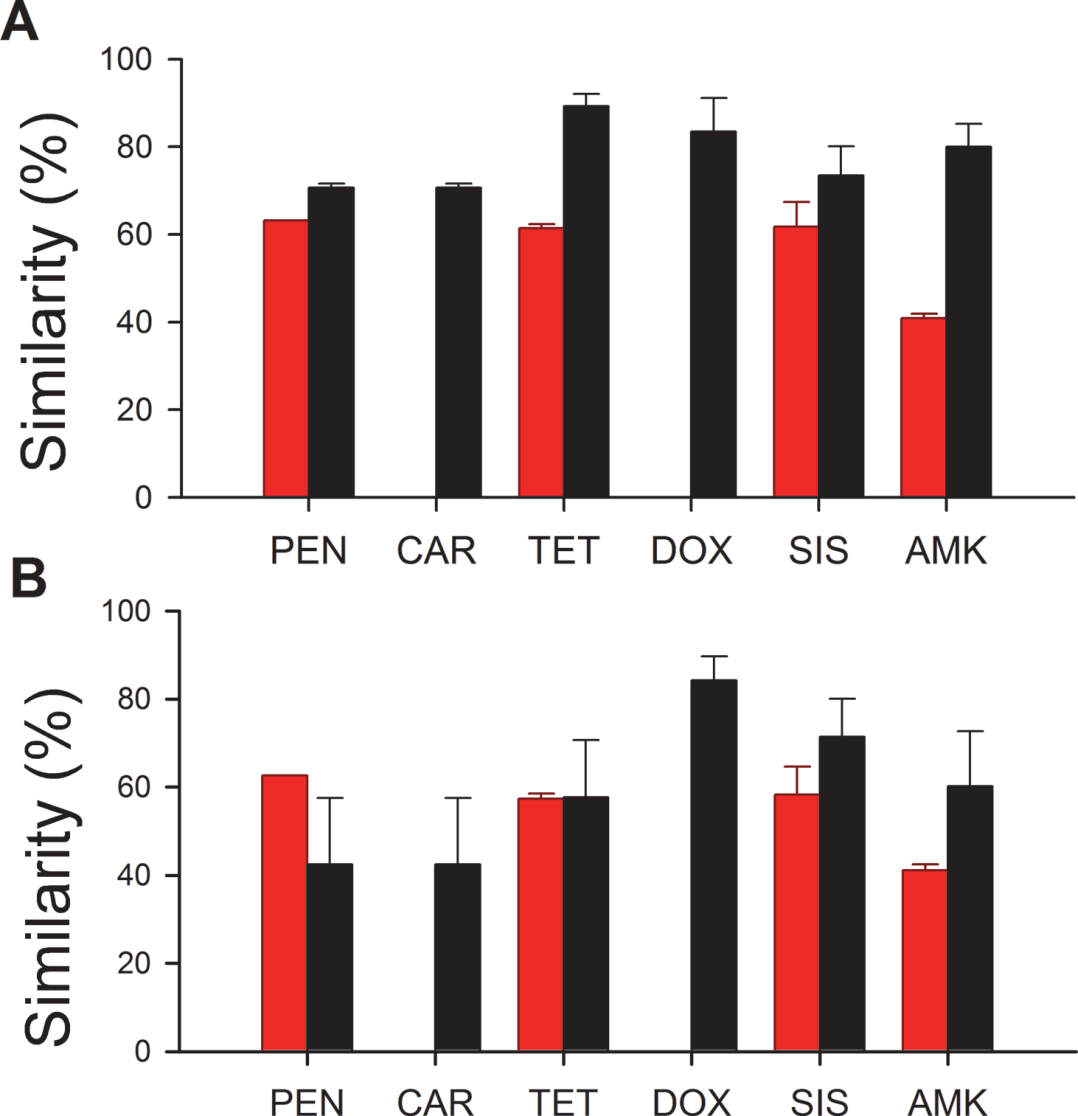
Nucleotide similarities of resistance genes isolated from ancient (red) and modern (black) bacteria with the closest homologous genes found in A) any bacteria or in B) a pathogenic bacteria.

Among ancient bacteria, we found eight unique resistance genes, which conferred resistance against four different antibiotics (Fig. 2; see Table G in S1 File for full description). More precisely, we observed resistance to all three native antibiotics, as well as resistance to one semi-synthetic antibiotic. By contrast, we found resistance to all six antibiotics among active layer bacteria, for a total of ten unique resistance genes (Fig. 2; see Table H in S1 File for full description). On average, the similarity between each resistance gene isolated from the active layer and the gene most related to it on GenBank [56] was higher than similarities between permafrost genes and their closest relatives *(F*_*(1*,*14)*_ *= 25.154; P < 0.001; Fig. 3A).* This difference disappeared when nucleotide similarity was compared with the closest related resistance gene harbored by a pathogenic strain, 63.2% for active layer genes and 54.9% for permafrost genes (*F*_(1,14)_ = 1.965; *P* > 0.10: Fig. 3B).

### Identifying host strains

Using diagnostic PCRs, we screened every bacterial strain for the presence of the resistance genes identified through our functional screen (Fig. 1C). Positive results were confirmed via re-sequencing, and we did not detect any false positives. In other words, the sequence of every amplified fragment corresponded to the predicted resistance genes. Among permafrost bacteria, we found that nine strains out of nineteen (47.3%) harbored at least one resistance gene (Table D in S1 File), and that eight strains out of twenty-five (32.0%) harbored resistance in the active layer (Table E in S1 File)(Fig. 1C). Among the active layer bacteria, we even found two isolates identified as *Stenotrophomonas* sp. carrying resistance to all three antibiotic classes (Fig. 1C; Table E in S1 File).

In bacteria associated with ancient permafrost, genes conferring resistance to tetracycline, sisomicin and amikacin were distributed among five different strains of Bacillaceae. (Fig. 1C; Table D in S1 File), a family of Gram-positive bacteria that produces over 150 different antimicrobials, including aminoglycosides [57]. In particular, resistance gene AMK_P_1 was most closely related to aminotransferases (Fig. 2 & Table G in S1 File), a group of enzymes associated with aminoglycoside biosynthesis in many bacterial species, including *Bacillus* [58]. *Bacillus* spp. are also known to possess a large number of resistance mechanisms for self-protection [57]. One such mechanism is a group of multidrug-efflux pumps that are related to TET_P_1, a tetracycline resistance gene found in three ancient *Bacillus* strains (Fig. 2 & Table D in S1 File; Fig. 1C). Therefore, resistance to tetracycline and aminoglycosides in *Bacillus* may reflect an evolutionary response to antimicrobials naturally produced within the genus.

We also found evidence for resistance evolution in response to environmental production of antibiotics. Gene PEN_P_1, conferring resistance to penicillin, was found in *Staphylococcus* sp. (Fig. 1C), an organism that does not produce β-lactam antibiotics [59]. Gene PEN_P_1 is most closely related to a penicillin acylase type II found in *Bacillus* (Fig. 2 & Table G in S1 File), an enzyme that hydrolyzes penicillin G into 6-aminopenicillanate [60]. The primary physiological role of the penicillin acylase in bacteria is believed to involve the utilization of aromatic amides as carbon sources; penicillins are in fact amidic compounds [61]. Even though penicillin acylases are used in the industry to develop antibiotics, the enzyme is not part of the normal β-lactams biosynthesis in fungi or bacteria [62]. Therefore, bacteria from the genus *Staphylococcus* most likely acquired the gene either to use β-lactams as a carbon source or to protect itself in response to the production of the antibiotics by other microorganisms in its environments. Although many members of the genus are widely distributed in natural environments [63], *Staphylococcus* are only rarely isolated from permafrost soils [28]. We confirmed that isolate Eur3 2.12 is physiologically different from *Staphylococcus* strains that might be possible sources of contamination by comparing Eur3 2.12’s growth at a range of temperatures. Consistent with its isolation from permafrost soils, strain Eur3 2.12 was able to grow between 5°C and 37°C (Table J in S1 File). When compared to laboratory standard strains of *S*. *aureus* and *S*. *epidermidis*, strain Eur3 2.12 showed higher growth at 5°C (Fig. B in S1 File), a trait shared by many psychrotolerant microorganisms [28].

### Resistance and cross-resistance

**Fig 4:**
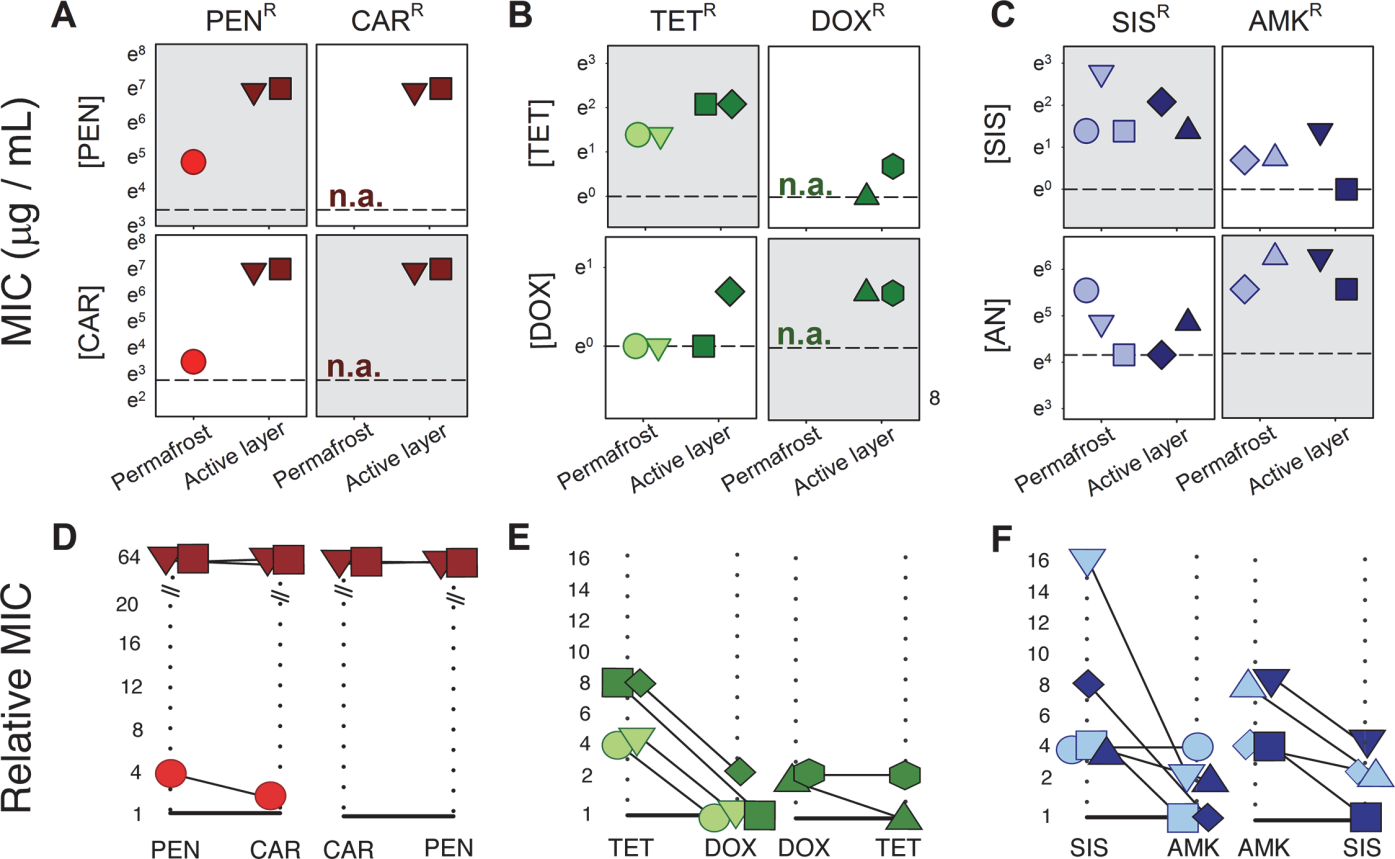
Resistance (A-C) and cross-resistance (D-F) levels of resistance genes isolated from ancient permafrost and its overlaying active layer. Each unique gene is depicted by a shape and color combination based on sampling site and antibiotic on which it was isolated (shown on top of panels): A) & D) **β**-lactams, penicillin (PEN) & carbenicillin (CAR); B) & E) tetracyclines, tetracycline (TET) & doxycycline (DOX); and C) & F) aminoglycosides, sisomicin (SIS) & amikacin (AMK). In panels A) to C), each point shows resistance to antibiotics indicated at left (measured as minimum inhibitory concentration, MIC). Grey panels indicate resistance levels to the drug in which genes were isolated, and white panels show cross-resistance to the other drug in the same class. Dashed line indicates MIC of control libraries. Panels D) to F) show slopegraphs of cross-resistance between antibiotics of a same family. The left axis represents relative resistance (MIC of the isolated genes / MIC of the control *E*. *coli* library) in the antibiotics where the gene was isolated. The right axis represents the relative fitness of the genes in the other antibiotic of the same class. Any slope that doesn’t go down to one on the right axis indicates some degree of cross-resistance.

Generally, resistance genes isolated from active layer bacteria conferred protection against higher concentrations of antibiotics than permafrost resistance genes (Fig. 4). Resistance genes from the active layers were also more likely to confer cross-resistance to other antibiotics (Fig. C in S1 File). We observed the largest difference in resistance levels between genes isolated from the active layer and the permafrost among β-lactam resistance genes (Fig. 4A). The penicillin resistance gene isolated from the permafrost conferred a two-fold increase in resistance, while genes PEN_AL_1 and PEN_AL_2 from the active layer conferred resistance to the highest concentrations of penicillin and carbenicillin we tested (MIC > 1024 μg/mL) (Fig. 4D). The latter are related to β-lactamases found in pathogenic bacteria: gene PEN_AL_1 is related to β-lactamases of the pseudomonads, including the opportunistic pathogen *Pseudomonas aeruginosa* (73.7% protein identity; Fig. D1 in S1 File) while gene PEN_AL_2 is most related to the L2 β-lactamases of *Stenotrophomonas maltohpilia* (68.3% protein identity; Fig. D2 in S1 File), an emerging opportunistic pathogen and a common etiological agent of septicemia [64]. As discussed above, gene PEN_P_1 was most related to penicillin acylases found in *Bacillus*, including several pathogenic isolates (Fig. E in S1 File).

We found a similar pattern for tetracycline resistance: genes isolated from the active layer conferred twice as much resistance as the genes isolated from the permafrost (Fig. 4B). Also, while tetracycline resistance genes found in the permafrost and the active layer were related to transporter/efflux pumps of various types (Figs. G & H in S1 File), resistance to the semi-synthetic doxycycline was observed only within the active layer (Fig. 4B). DOX-AL_1 and DOX_AL_2 were most closely related to acyltransferases involved in the lipolipid biosynthesis of *Stenotrophomonas* sp. (Fig. 2 & Table H in S1 File), likely contributing to the bacterium’s intrinsic resistance against multiple antibiotics [65,66]. Acyltransferases were previously associated with intrinsic resistance in pathogenic mycobacteria and with the inactivation of chloramphenicol in Gram-negative bacteria [67]. Again, cross-resistance between tetracycline and doxycycline was only observed in resistance genes isolated from the active layer (Fig. 4E).

In contrast with β-lactams and tetracyclines, we did not find a general trend in resistance levels among aminoglycoside antibiotic resistance genes. The three sisomicin resistance genes found in ancient soil bacteria and the two genes found among contemporary bacteria showed similar levels of resistance (Fig. 4C; Fig. 2 & Table G in S1 File). Genes SIS_P_2, found in the permafrost (Fig. 2; Table G in S1 File), and SIS_AL_2, found in the active layer (Fig. 2 & Table H in S1 File), were both related to aminoglycoside N6’-acetyltransferase, or AAC(6’), one of the most studied families of resistance genes against aminoglycosides [68]. Both genes present a high degree of divergence at the amino acid level when compared to the most related genes found in GenBank, 51.8% and 52.3% global protein identity with the closest related gene respectively (Fig. H1 & H2 in S1 File), suggesting that this resistance mechanism had diversified long before the anthropogenic use of antibiotics [68]. In addition to two sisomicin resistance genes conferring cross-resistance to semi-synthetic antibiotic amikacin (Fig. 4F), we isolated two genes conferring resistance uniquely to the antibiotic within the permafrost (Fig. 4C & F; Fig. 2 & Table G in S1 File). While amikacin-resistance genes from the permafrost showed a lower level of similarity to their closest homologs (Fig. H in S1 File) than the amikacin-resistance genes found in active layer bacteria (Fig. I in S1 File), both genes conferred cross-resistance to sisomicin when tested in planktonic culture (Fig. 4F).

### Environmental distribution of resistance genes

Finally, we used a comparative metagenomic strategy to examine the distribution of each resistance gene in microbial surveys of the active layer and the permafrost 2-m subsection (Table J in S1 File). Most resistance genes isolated from permafrost bacteria were in fact isolated from the latter subsection. We found homologous sequences to every resistance gene in both active layer and permafrost communities, except for the two β-lactamases, which were absent from the 2-m permafrost survey (Table 2). We also found that the vast majority of resistance genes isolated from permafrost and active layer shared significant identity to genes found in both soil and marine environments (Table K in S1 File), but were less frequent or absent in gut microbiomes (Table L in S1 File; Fig. J in S1 File). Finally, every resistance gene isolated in our study also showed some levels of similarity to genes found in pathogenic bacteria (Figs. D-I in S1 File).

**Table 2:** Number of sequences homologous to functional resistance genes in metagenomic surveys of the Canadian high Arctic.

	**PEN_P_1**	**TET_P_1**	**TET_P_2**	**SIS_P_1**	**SIS_P_2**	**SIS_P_3**	**AMK_P_1**	**AMK_P_2**	**PEN_AL_1**	**PEN_AL_1**	**TET_AL_1**	**TET_AL_2**	**SIS_AL_1**	**SIS_AL_2**	**AMK_AL_1**	**AMK_AL_2**	**DOX_AL_1**	**DOX_AL_2**
**Samples**																		
Active Layer	94	34	43	6	4	1	297	1	88	4	44	43	40	700	3	49	1	57
2-m permafrost	9	12	21	1	1	1	25	1	0	0	12	10	4	123	2	12	0	5

Metagenomic surveys data is described in Steven B, Pollard WH, Greer CW, & Whyte LG (2008) Microbial diversity and activity through a permafrost/ground ice core profile from the Canadian high Arctic. *Environ Microbiol* 10(12):3388–3403.

## Discussion

In this study, we demonstrate that diverse functional antibiotic resistance mechanisms existed in bacteria at least 5,000 years ago. By conducting a functional metagenomics screen of bacteria isolated from ancient permafrost, we identified genes conferring resistance to four different antibiotics, covering three major classes of antimicrobials used in modern medicine. Many of the resistance genes isolated in our study were highly similar to resistance genes found in pathogenic bacteria today (Figs. C-H). Furthermore, functional resistance genes were found both in bacterial genera known to produce antimicrobials as well as bacteria that are not normally associated with antimicrobial-production. Taken together these results support the hypothesis that a reservoir of resistance genes existed in a range of bacteria species prior to the discovery of antibiotics by Sir Alexander Fleming [24–26,33,69] and contribute to a growing body of evidence demonstrating that antibiotic resistance evolved alongside antibiotic production in the natural environment [6,33,35].

In a single sampling of the Canadian high Arctic permafrost, we found eight different resistance genes that encompass three broad classes of resistance mechanisms. More specifically, we found three resistance genes related to efflux pumps or transporters, two degrading enzymes and three genes related to membrane modification or synthesis. This diversity in functional resistance genes could help explain the rapid evolution of resistance against modern-day antibiotics, including semi-synthetic antibiotics developed and synthesized in the laboratory. Indeed, among the five genes conferring resistance to aminoglycosides, four provided resistance or cross-resistance against amikacin. This antibiotic was the first semi-synthetic aminoglycoside used in medicine and was specifically designed to counter resistance to native aminoglycosides such as streptomycin and sisomicin [44]. In fact, one of the amikacin resistance genes we isolated was related to aminoglycoside-N6’-acetyltransferases found in modern *Citrobacter* spp. and *Salmonella enterica* (Fig. G2 in S1 File). Therefore, it is perhaps not surprising that amikacin resistance was quickly discovered in clinical isolates of *Salmonella* and other *Enterobacteriaceae* within a year of the antibiotic’s introduction [70,71].

The above results suggest that exhaustive resistance screening strategies could help predicting the success of new antimicrobial molecules [72]. Information on the frequency and the diversity of functional resistance genes in natural microbial communities prior to the introduction of a new drug can tell us whether the drug has the potential to remain effective against pathogenic bacteria for significant periods of time. For example, the first acquired resistance gene against quinolones, a fully synthetic class of antibiotics for which distant analogues exist in the wild [73], was discovered only recently in clinical isolates [74,75]. Even though sequences homologous to the acquired resistance gene (*qrn)* were found in the genomes of many Gram-negative and Gram-positive bacteria [76], functional resistance to the synthetic antibiotic evolved in clinical populations mainly through the acquisition of point mutations in the genes encoding either of the two type IIA topoisomerases targeted, DNA gyrase and DNA topoisomerase IV [77]. Therefore, resistance to quinolones in clinical strains remained manageable for more than two decades after the introduction of the antibiotic class [78].

We also found that resistance genes associated with bacteria isolated in the active layer generally conferred higher levels of resistance than resistance genes isolated from the permafrost. Increases in resistance levels observed in soil microbiomes [79,80] or in clinical isolates [11] have been considered as evidence for the impact of antibiotic use on microbial communities. In our study, the difference in resistance levels is also associated with changes in community composition. For example, there seems to be a slight bias towards spore-forming bacteria in the permafrost community. Furthermore, there is growing evidence that subpopulations of microorganisms in the permafrost constitute active microbial ecosystem rather than “ancient” frozen microbial survivors [39,81]. Therefore, whether antibiotic resistance level changes in permafrost result from changes in community composition, local ecological interactions or are the consequences of anthropogenic antibiotic use remains to be tested.

Although the study of culturable bacteria enabled us to accurately identify the taxa associated with antibiotic resistance in ancient permafrost, it likely underestimates the total number of resistance genes in our samples. The use of a specific host and of a high copy number plasmid also likely affected the identity of the resistance genes found in this study [82]. Indeed, we expect that resistance genes that are distantly related to *E*. *coli* or more generally to Gram-negative bacteria might be more difficult to detect. Still, the use of functional screens is a powerful way to detect and confirm the phenotype of antibiotic resistance genes in microbial communities [3,7,27]. While using a Gram-negative host enables us to detect resistance genes that are more likely to be relevant in pathogenic bacteria such as *E*. *coli*, *P*. *aeruginosa* and *Salmonella*, the amplification of bacterial genes on high copy number plasmids can inform us on the possible effect of gene duplication or on the transfer of a gene to a plasmid [83].

The existence of a resistance reservoirs in the environment can greatly accelerate the evolution of multidrug-resistant bacteria [7]. For this reason, it is crucial to take into account the extensive diversity of antibiotic resistance genes found in microbial populations when developing or deploying new antibiotic strategies. Future studies of ancient permafrost soils including total metagenomics DNA and additional sampling will allow us to study the temporal and spatial distribution of antibiotic resistance genes and the possible impacts of human activity on the microbial world. For instance, it would be interesting to know whether the diversity of antibiotic resistance genes as well as the prevalence of resistance genes followed similar trends over time. While diversity can be measure as the total number of resistance genes identified in a library, prevalence could be measured as the total number of growing colonies on selective plates given the total amount of DNA used to build the library. Finally, whole-genome analyses of bacteria isolated from ancient soils should shed a new light on the role of horizontal gene transfer in the evolution of antibiotic resistance and emerging diseases in general.

## Supporting Information

S1 File Supporting figures and tables. **Figure A:** Sampling depth of resistance conferring inserts. **Figure B:** Temporal growth profile of strains Eur3 2.12, *Staphylococcus aureus* strain MCD01 and *Staphylococcus epidermidis* strain MCD02 at 5°C. **Figure C:** Resistance and cross-resistance levels conferred by inserts isolated from the permafrost and the active layer of single core collected from the Canadian high Arctic. **Figure D:** Phylogenetic distribution of full-length gene products encoding resistance to beta-lactams isolated from the Canadian high Artic active layer soil. **Figure E:** Phylogenetic distribution of full-length gene products encoding resistance to beta-lactams isolated from the Canadian high Artic permafrost. **Figure F:** Phylogenetic distribution of full-length gene products encoding resistance to tetracycline isolated from the Canadian high Arctic permafrost. **Figure G:** Phylogenetic distribution of full-length gene products encoding resistance to tetracycline isolated from the Canadian high Arctic active layer soil. **Figure H:** Phylogenetic distribution of full-length gene products encoding resistance to aminoglycoside isolated from the Canadian high Arctic permafrost. **Figure I:** Phylogenetic distribution of full-length gene products encoding resistance to aminoglycoside isolated from the Canadian high Arctic active layer soil. **Figure J:** Abundance of putative resistance genes and related proteins at the sampling sites and other metagenomes. **Table A:** List of strains, plasmids and primers used for library construction. **Table B:** Primers used to identify the permafrost bacteria strain(s) harboring each resistant inserts. **Table C:** Primers used to identify the active layer bacteria strain(s) harboring each resistant inserts. **Table D:** List of bacteria strains isolated from the permafrost and associated resistance genes. **Table E:** List of bacteria strains isolated from active layer and associated resistance genes. **Table F:** Numbers of antibiotic resistant clones sequenced and unique resistance genes found from a functional analysis of the permafrost and the active layer of the Canadian high Arctic. **Table G:** Resistance genes identified using metagenomic functional selections from Canadian High Arctic permafrost. **Table H:** Resistance genes identified using metagenomic functional selections from Canadian High Arctic active layer. **Table I:** List of environmental microbiomes used for studying the distribution of each resistant insert. **Table J:** Growth profile of bacterial isolate at different temperatures. **Table K:** Number of significant BLASTP hits across environmental microbiomes. **Table L:** Number of significant BLASTP hits across gut microbiomes.
(DOC)
