# Supplementary material for: Functional Characterization of Bacteria Isolated from Ancient Arctic Soil Exposes Diverse Resistance Mechanisms to Modern Antibiotics
Source: PLoS One. 2015 Mar 25;10(3):e0069533. doi: 10.1371/journal.pone.0069533 (PMC4373940; doi:10.1371/journal.pone.0069533)
Supplement: S1 File — Figure A: Sampling depth of resistance conferring inserts. Figure B: Temporal growth profile of strains Eur3 2.12, Staphylococcus aureus strain MCD01 and Staphylococcus epidermidis strain MCD02 at 5°C. Figure C: Resistance and cross-resistance levels conferred by inserts isolated from the permafrost and the active layer of single core collected from the Canadian high Arctic. Figure D: Phylogenetic distribution of full-length gene products encoding resistance to beta-lactams isolated from the Canadian high Artic active layer soil. Figure E: Phylogenetic distribution of full-length gene products encoding resistance to beta-lactams isolated from the Canadian high Artic permafrost. Figure F: Phylogenetic distribution of full-length gene products encoding resistance to tetracycline isolated from the Canadian high Arctic permafrost. Figure G: Phylogenetic distribution of full-length gene products encoding resistance to tetracycline isolated from the Canadian high Arctic active layer soil. Figure H: Phylogenetic distribution of full-length gene products encoding resistance to aminoglycoside isolated from the Canadian high Arctic permafrost. Figure I: Phylogenetic distribution of full-length gene products encoding resistance to aminoglycoside isolated from the Canadian high Arctic active layer soil. Figure J: Abundance of putative resistance genes and related proteins at the sampling sites and other metagenomes. Table A: List of strains, plasmids and primers used for library construction. Table B: Primers used to identify the permafrost bacteria strain(s) harboring each resistant inserts. Table C: Primers used to identify the active layer bacteria strain(s) harboring each resistant inserts. Table D: List of bacteria strains isolated from the permafrost and associated resistance genes. Table E: List of bacteria strains isolated from active layer and associated resistance genes. Table F: Numbers of antibiotic resistant clones sequenced and unique resistance genes found from a [file pone.0069533.s001.doc]

**SUPPlementary Information**

**Functional characterization of bacteria from ancient soil exposes resistance mechanisms to modern antibiotics**

Gabriel G. Perron^1,2,3*^, Lyle Whyte^4^, Peter J. Turnbaugh^1,5^, Jacqueline Goordial^4^, William P. Hanage^6^, Gautam Dantas^7,8^ & Michael M. Desai^1,2,9*^

1-FAS Center for Systems Biology, Harvard University, 52 Oxford Street, Cambridge, Massachusetts, USA 02138.

2-Department of Evolutionary and Organismic Biology, Harvard University, 52 Oxford Street, Cambridge, Massachusetts, USA 02138.

3-Biology Program, Bard College, 30 Campus Road, Annandale-on-Hudson, NY 12504.

4-Department of Natural Resource Sciences, McGill University, Macdonald Campus, 21,111 Lakeshore, Ste-Anne-de-Bellevue, Quebec, Canada H9X 3V9.

5-Department of Microbiology and Immunology, Hooper Foundation, University of California San Francisco, 513 Parnassus Ave, San Francisco, CA 94143, USA.

6-Department of Epidemiology, Harvard School of Public School, 677 Huntington Avenue, Boston, Massachusetts, USA 02115.

7-Center for Genome Sciences and Systems Biology, Washington University School of Medicine, 4444 Forest Park Avenue, St. Louis, Missouri, USA 63108.

8-Department of Pathology and Immunology, Washington University School of Medicine, 4444 Park Forest Avenue, St. Louis, Missouri, USA 63108

9-Department of Physics, Harvard University, Cambridge Massachusetts, USA 02138.

*To whom correspondence should be addressed: (GGP) gperron@bard.edu / 617-866-0464; (MMD) mmdesai@fas.harvard.edu / 617-496-3613.

**Figure A.** **Sampling depth of resistance conferring inserts**. For the permafrost library **1**) and active layer library **2**), we isolated and sequenced five resistant clones for each antibiotic where growth was observed. The figure depicts the total number of unique sequences as we randomly sample all the inserts selected in this study.

**1)**


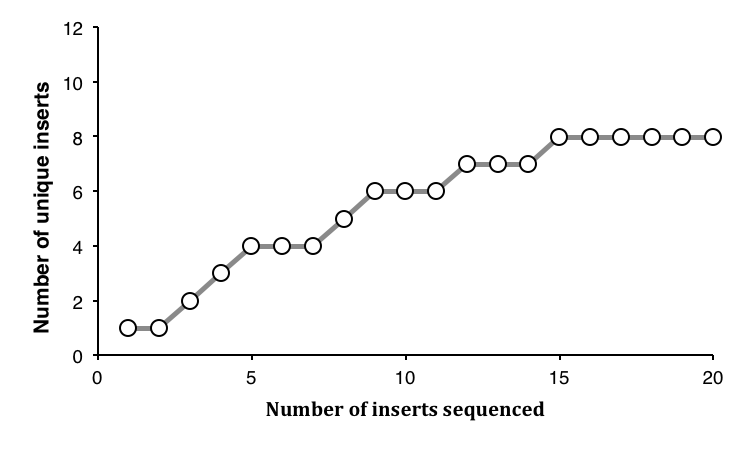


**2)**


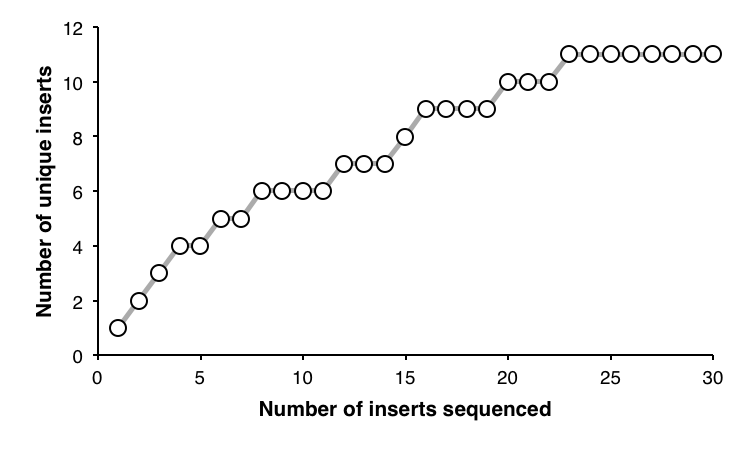


**Figure B**: Temporal growth profile of strains Eur3 2.12, *Staphylococcus aureus* strain MCD01 and *Staphylococcus epidermidis* strain MCD02 at 5°C.

**Figure C.** **Resistance and cross-resistance levels conferred by inserts isolated from the permafrost and the active layer of single core collected from the Canadian high Arctic**. Data is shown for six antibiotics belonging to three different antibiotic classes: (red) beta-lactams; (green) tetracyclines; and (blue) aminoglycosides. Each insert is depicted by a single shape and color combination. Antibiotics are as follows: penicillin (PEN, native), carbenicillin (CAR, semi-synthetic), tetracycline (TET, native), doxycycline (DOX, semi-synthetic), sisomicin (SIS, native), and amikacin (AMK, semi-synthetic). Each point shows the resistance to the antibiotic indicated at left (measured as minimum inhibitory concentration, MIC) that is conferred by a gene isolated from a screen using the antibiotic shown at top. Grey panels thus indicate the resistance level to the drug used to isolate the gene, and white panels show cross-resistance provided by these genes to the other drugs. Dashed line indicates the MIC of the control library. No significant cross-resistance across different drug classes was observed.

*
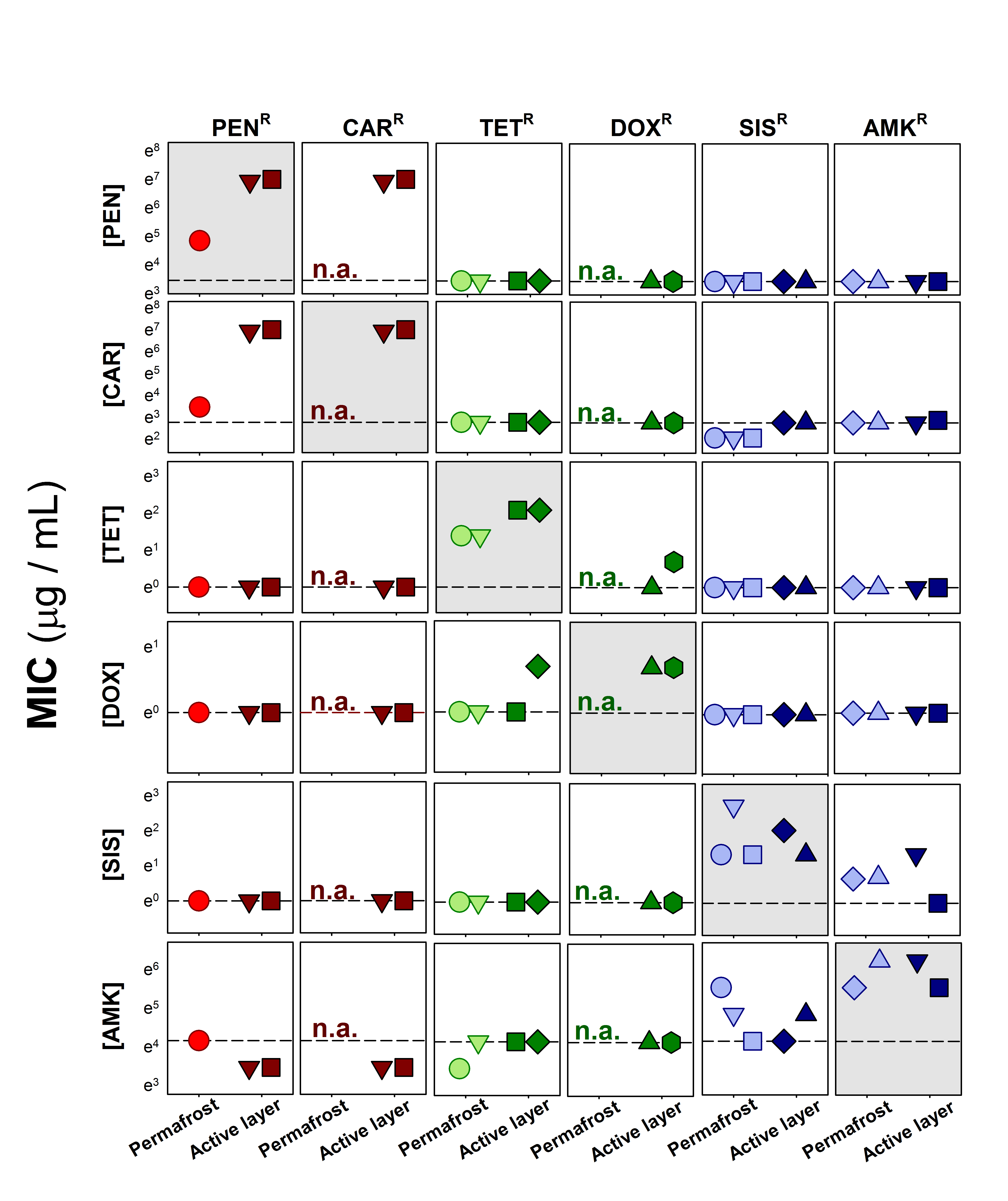
*

**Figure D**. **Phylogenetic distribution of full-length gene products encoding resistance to beta-lactams isolated from the Canadian high Artic active layer soil**: **1)** PEN/CAR_AL_1 and **2)** PEN/CAR_AL_2. To give an overview of the related genes in Genbank, every fourth sequence of the top 100 hits using tblastx were included in the phylogeny. The tip of the branches are identified with the sequence accession number in Genbank, the species of the organism, and when available the source (color code: red is pathogen; dark red is human activity; brown is animal; green is plant; blue is aquatic; and black is soil). Unrooted phylogenetic trees were generated from ClustalW alignment, and a consensus tree (70% of 10,000 bootsraps) was constructed using the neighbor-joining algorithm. Tree branch lengths are proportional to relative sequence identity, and the scale bar is in fixed amino acid substitution per sequence position.

**1)**


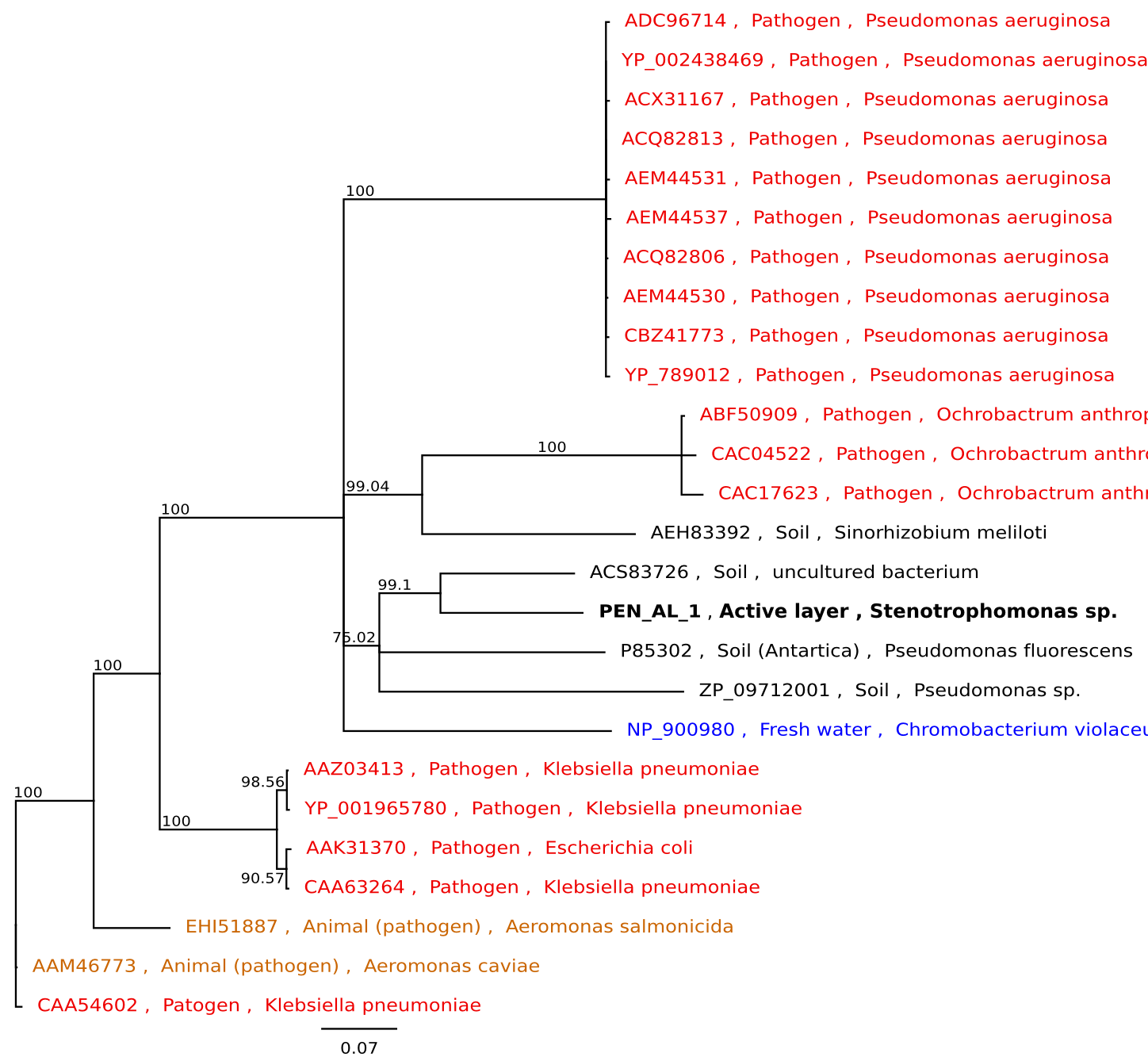


**2)**


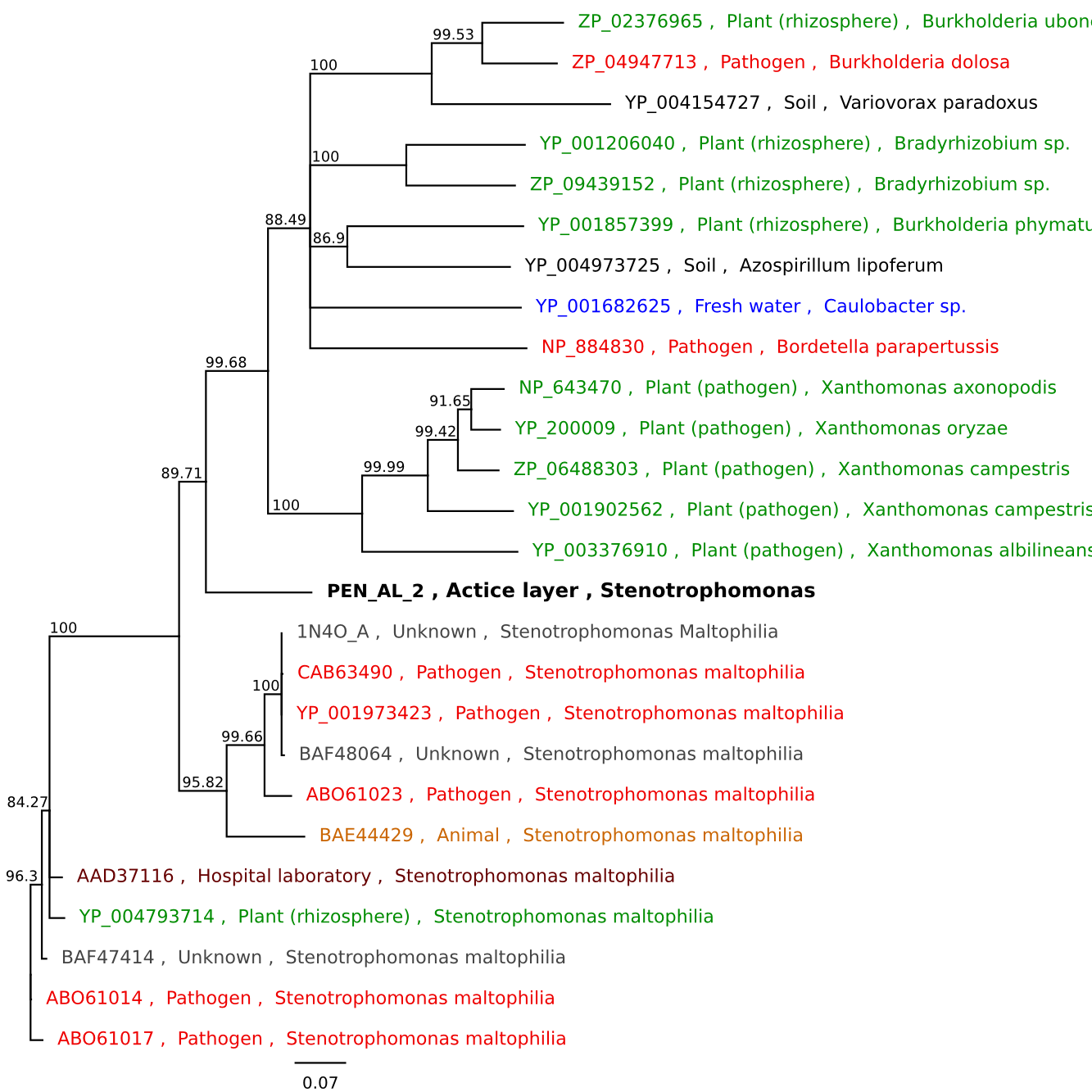


**Figure E**. **Phylogenetic distribution of full-length gene products encoding resistance to beta-lactams isolated from the Canadian high Artic permafrost**. To give an overview of the related genes in Genbank, every fourth sequence of the top 100 hits using tblastx were included in the phylogeny. The tip of the branches are identified with the sequence accession number in Genbank, the species of the organism, and when available the source (color code: red is pathogen; dark red is human activity; brown is animal; green is plant; blue is aquatic; and black is soil). Unrooted phylogenetic trees were generated from ClustalW alignment, and a consensus tree (70% of 10,000 bootsraps) was constructed using the neighbor-joining algorithm. Tree branch lengths are proportional to relative sequence identity, and the scale bar is in fixed amino acid substitution per sequence position.


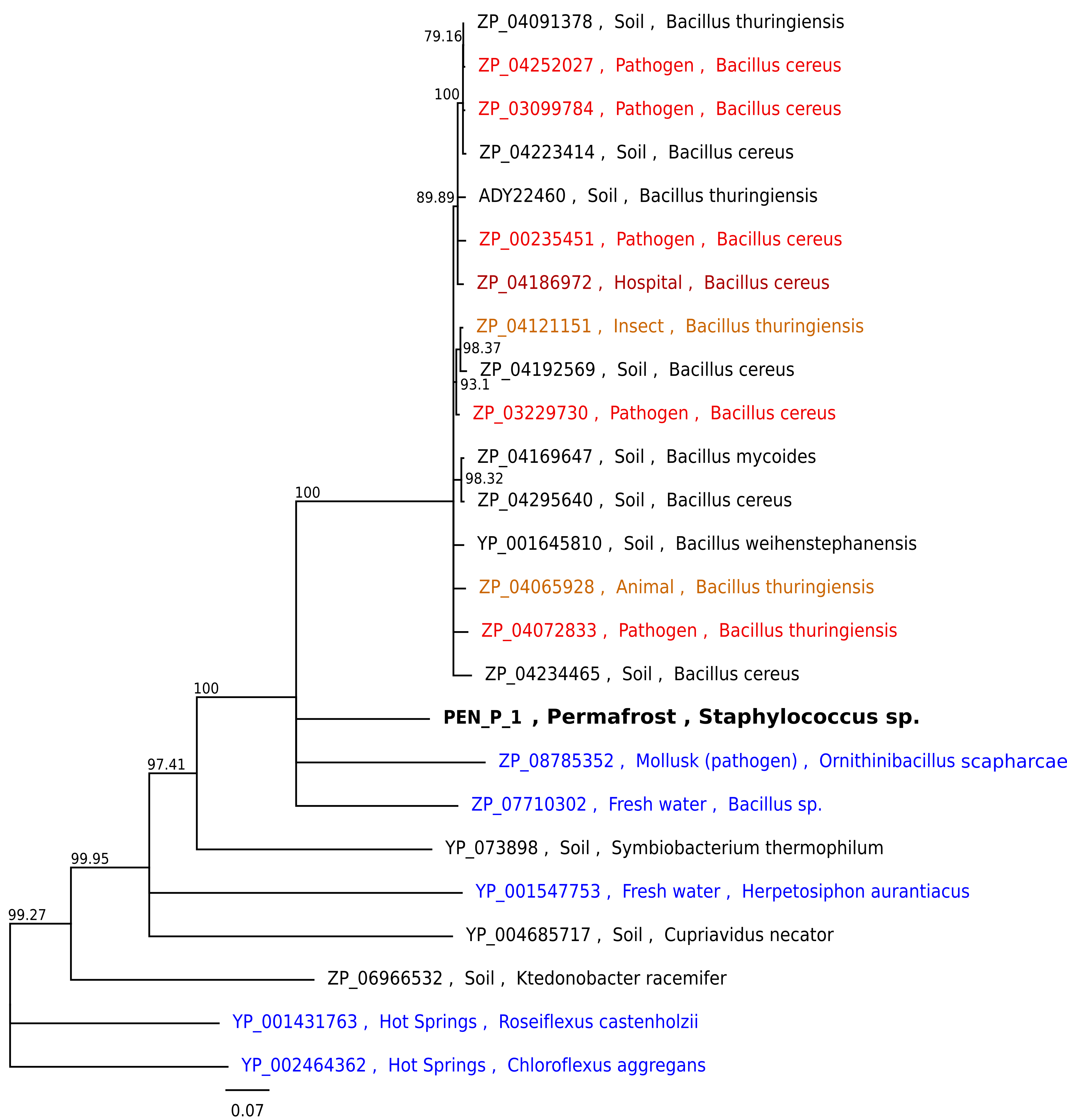


**Figure F**. **Phylogenetic distribution of full-length gene products encoding resistance to tetracycline isolated from the Canadian high Arctic permafrost**: **1)** TET_P_1 and **2)** TET_P_2. To give an overview of the related genes in Genbank, every fourth sequence of the top 100 hits using tblastx were included in the phylogeny. The tip of the branches are identified with the sequence accession number in Genbank, the species of the organism, and when available the source (color code: red is pathogen; dark red is human activity; brown is animal; green is plant; blue is aquatic; and black is soil). Unrooted phylogenetic trees were generated from ClustalW alignment, and a consensus tree (70% of 10,000 bootsraps) was constructed using the neighbor-joining algorithm. Tree branch lengths are proportional to relative sequence identity, and the scale bar is in fixed amino acid substitution per sequence position.

**1**)


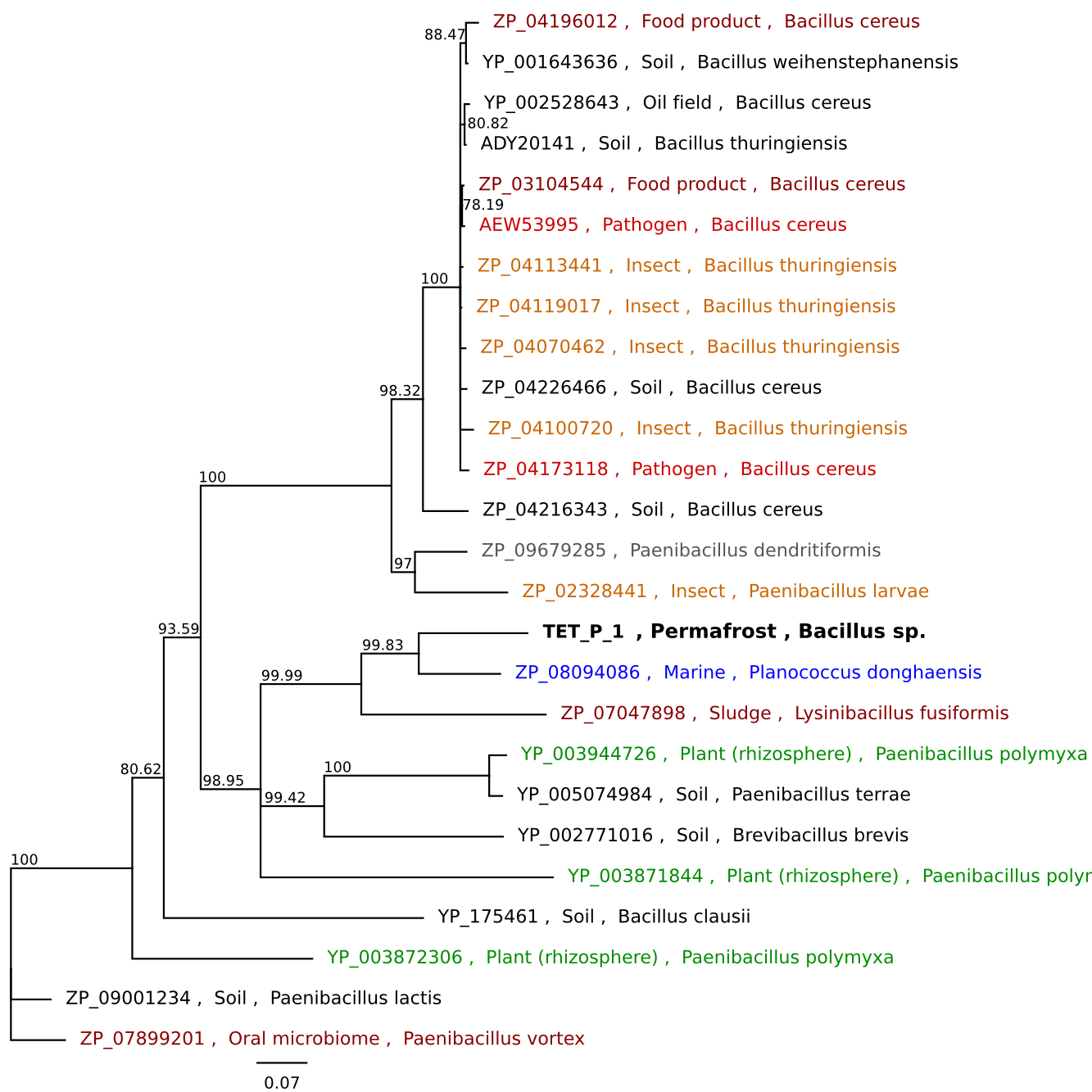


**2)**


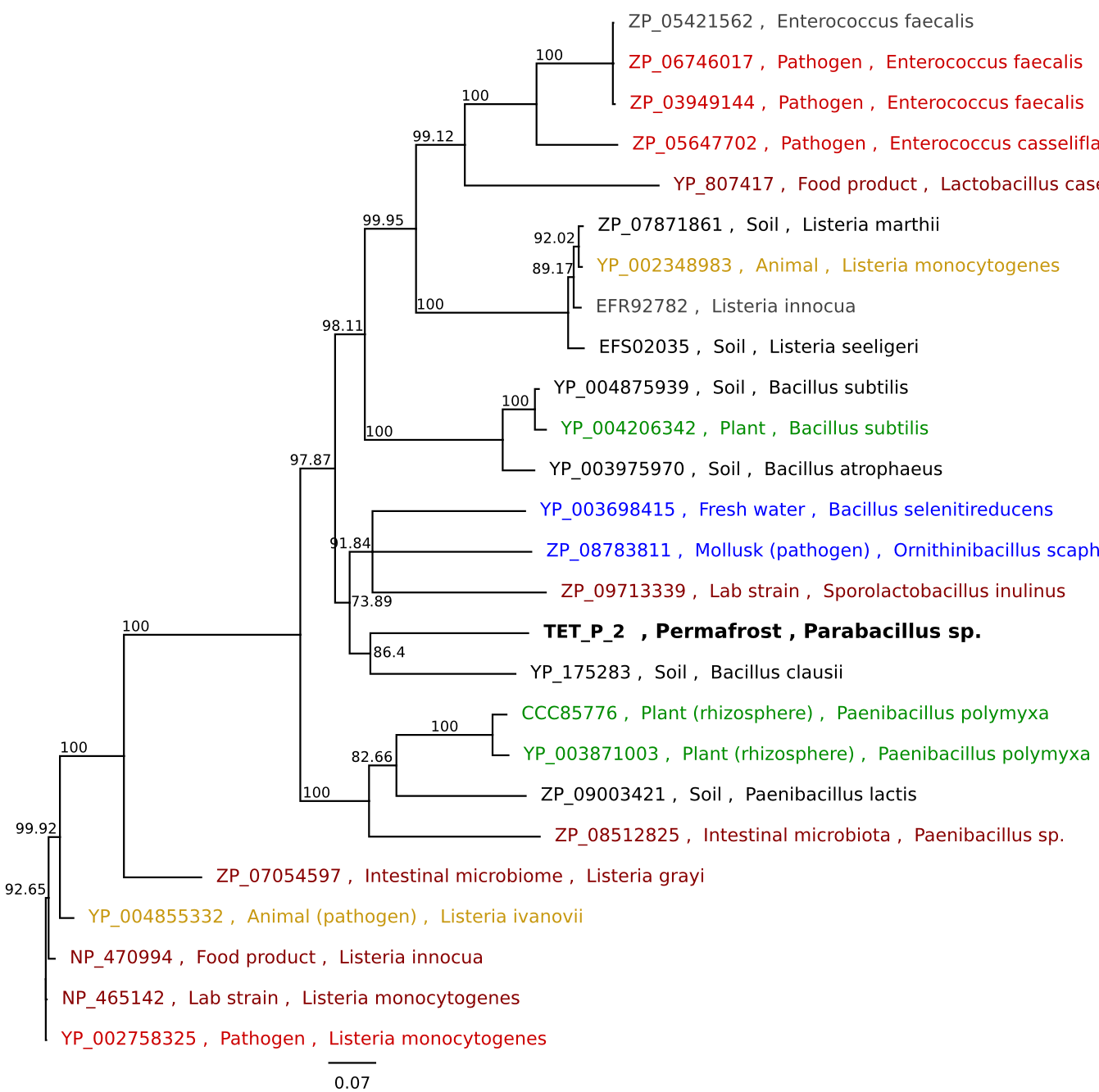


**Figure G**. **Phylogenetic distribution of full-length gene products encoding resistance to tetracycline isolated from the Canadian high Arctic active layer soil**: **1)** TET_AL_1; **2)** TET_AL_2; **3)** DOX_AL1; and **4)** DOX_AL2. To give an overview of the related genes in Genbank, every fourth sequence of the top 100 hits using tblastx were included in the phylogeny. The tip of the branches are identified with the sequence accession number in Genbank, the species of the organism, and when available the source (color code: red is pathogen; dark red is human activity; brown is animal; green is plant; blue is aquatic; and black is soil). Unrooted phylogenetic trees were generated from ClustalW alignment, and a consensus tree (70% of 10,000 bootsraps) was constructed using the neighbor-joining algorithm. Tree branch lengths are proportional to relative sequence identity, and the scale bar is in fixed amino acid substitution per sequence position.

**1)**


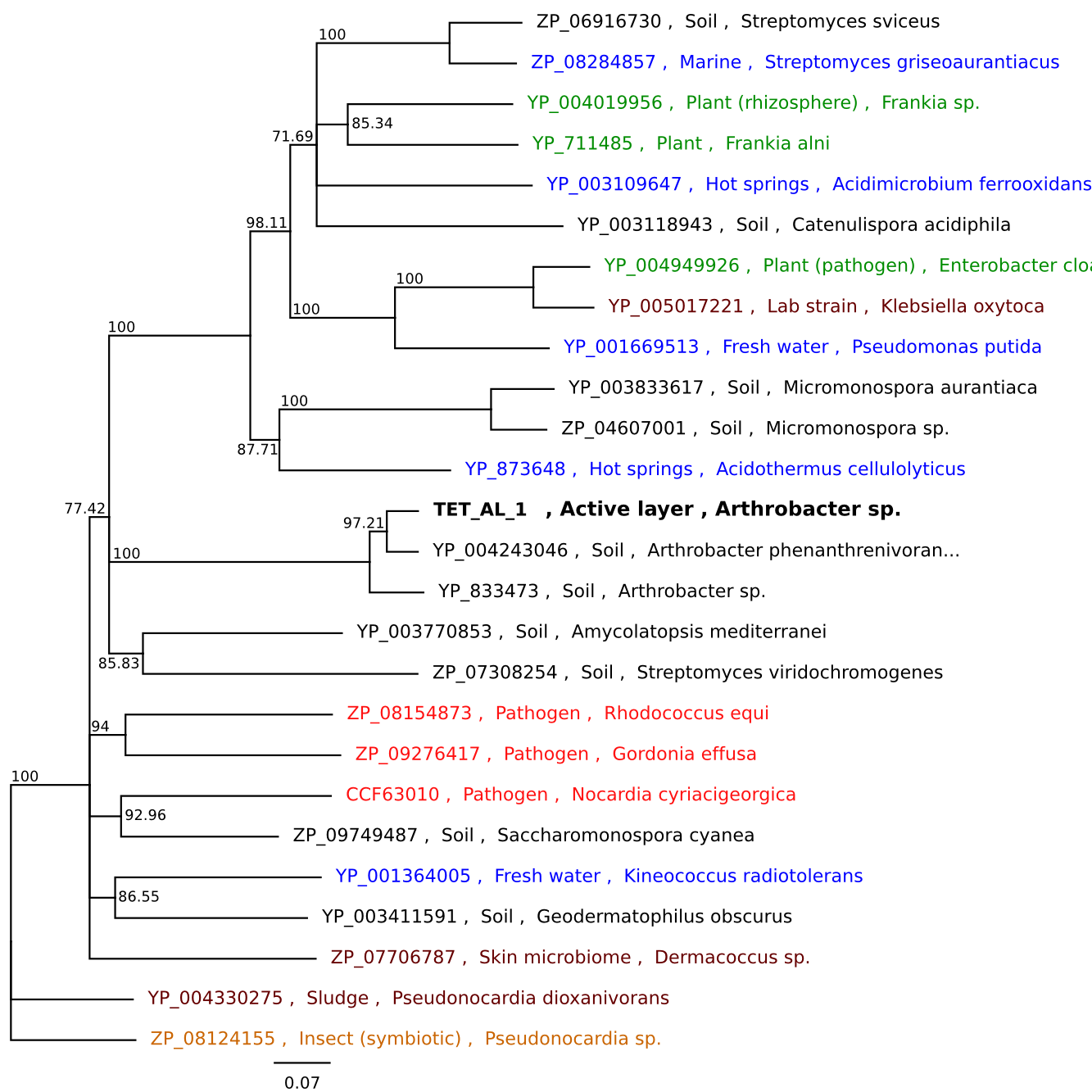


**2)**


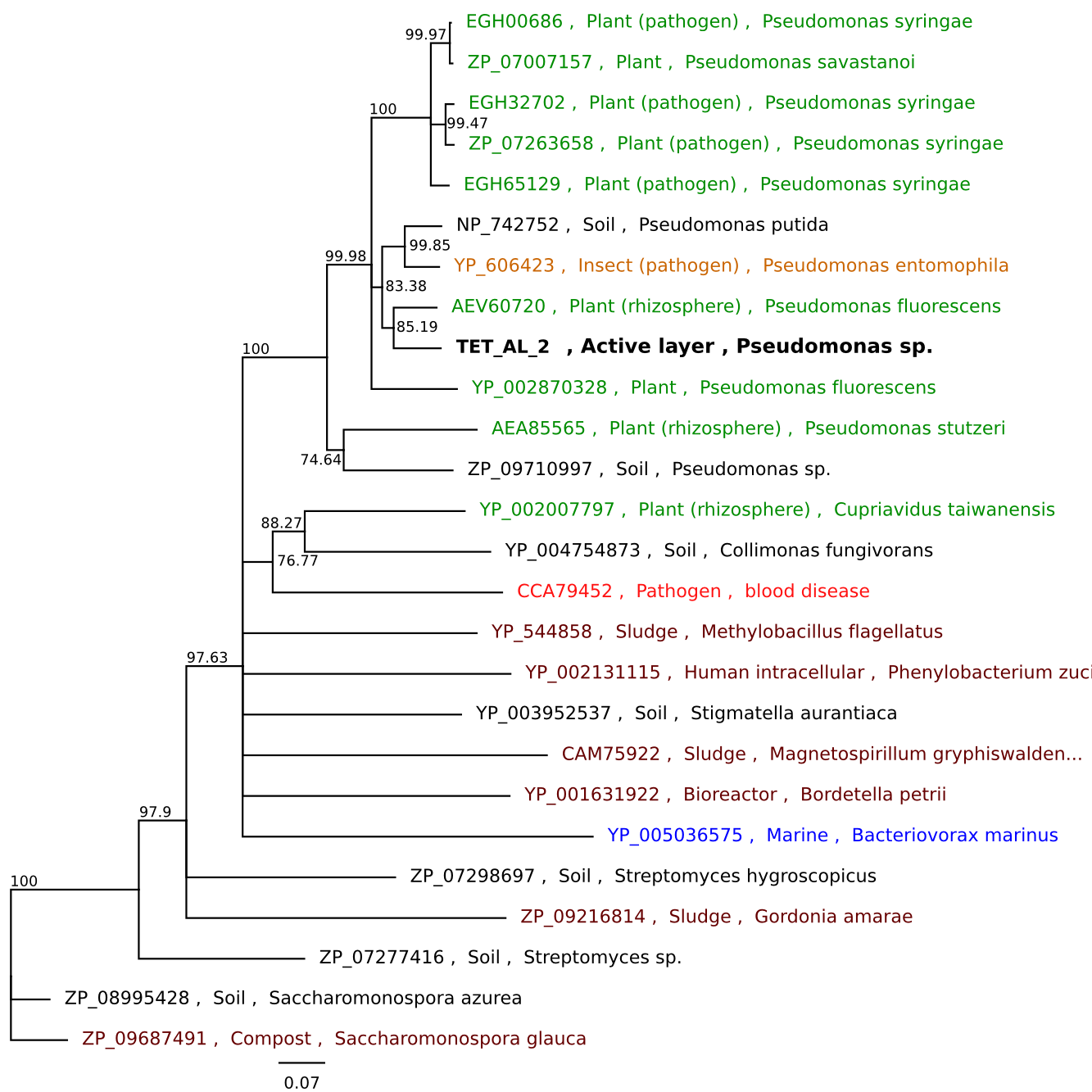


**3)**


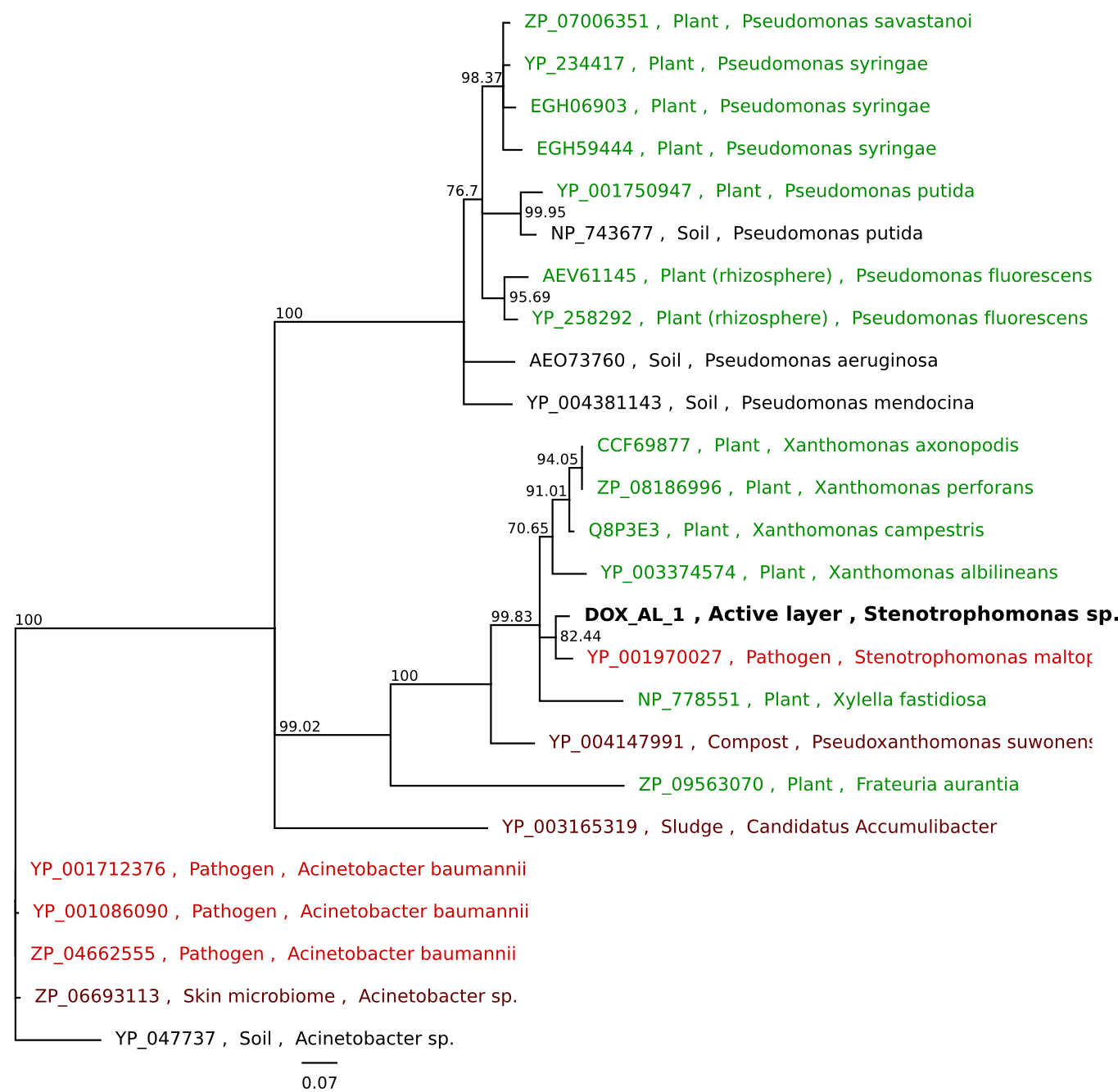


**4)**

**
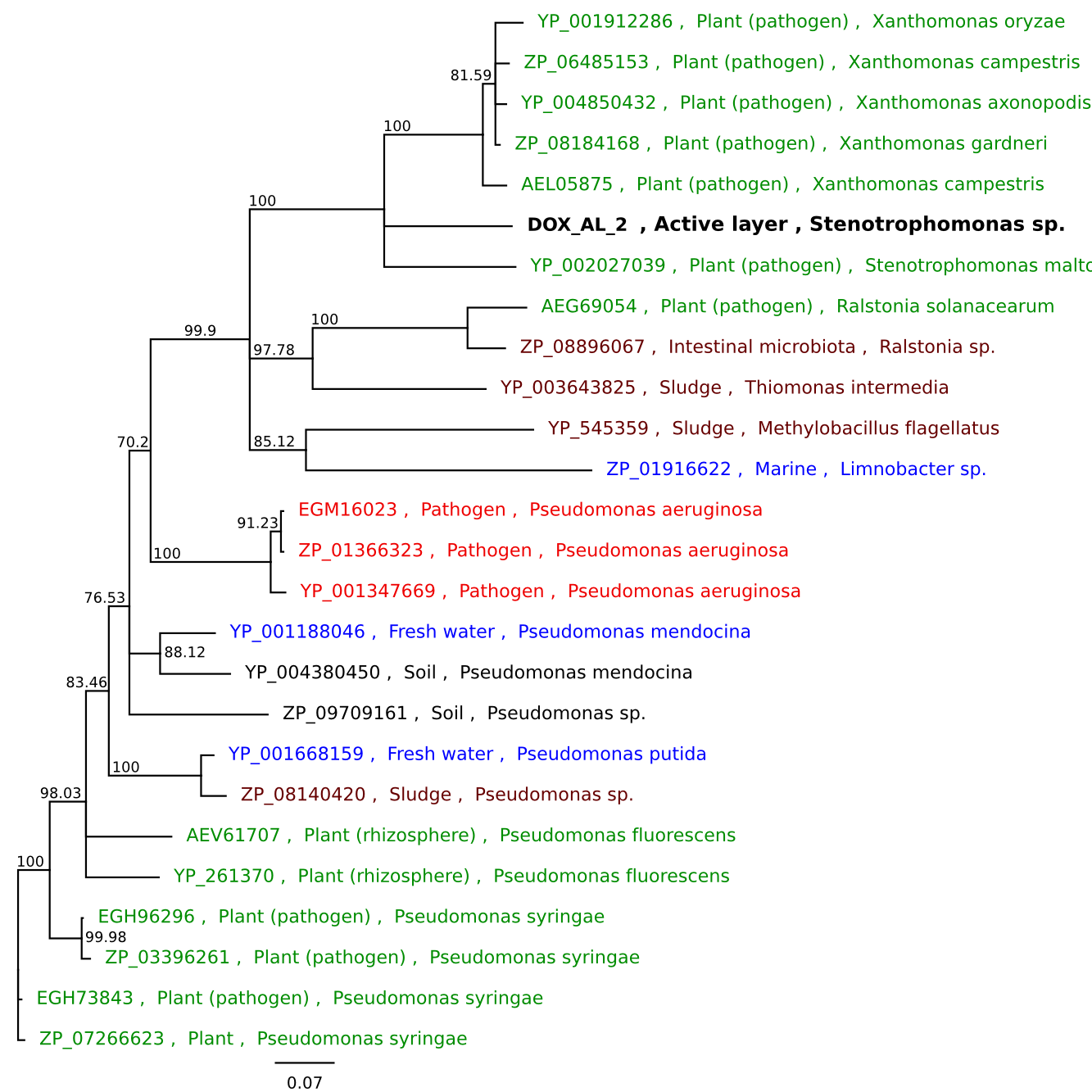
**

**Figure H**. **Phylogenetic distribution of full-length gene products encoding resistance to aminoglycoside isolated from the Canadian high Arctic permafrost**: **1)** SIS_P_1; **2)** SIS_P_2; **3)** SIS_P_3; **4)** AMK_P_1; **5)** AMK_P_2. To give an overview of the related genes in Genbank, every fourth sequence of the top 100 hits using tblastx were included in the phylogeny. The tip of the branches are identified with the sequence accession number in Genbank, the species of the organism, and when available the source (color code: red is pathogen; dark red is human activity; brown is animal; green is plant; blue is aquatic; and black is soil). Unrooted phylogenetic trees were generated from ClustalW alignment, and a consensus tree (70% of 10,000 bootsraps) was constructed using the neighbor-joining algorithm. Tree branch lengths are proportional to relative sequence identity, and the scale bar is in fixed amino acid substitution per sequence position.

**1)**


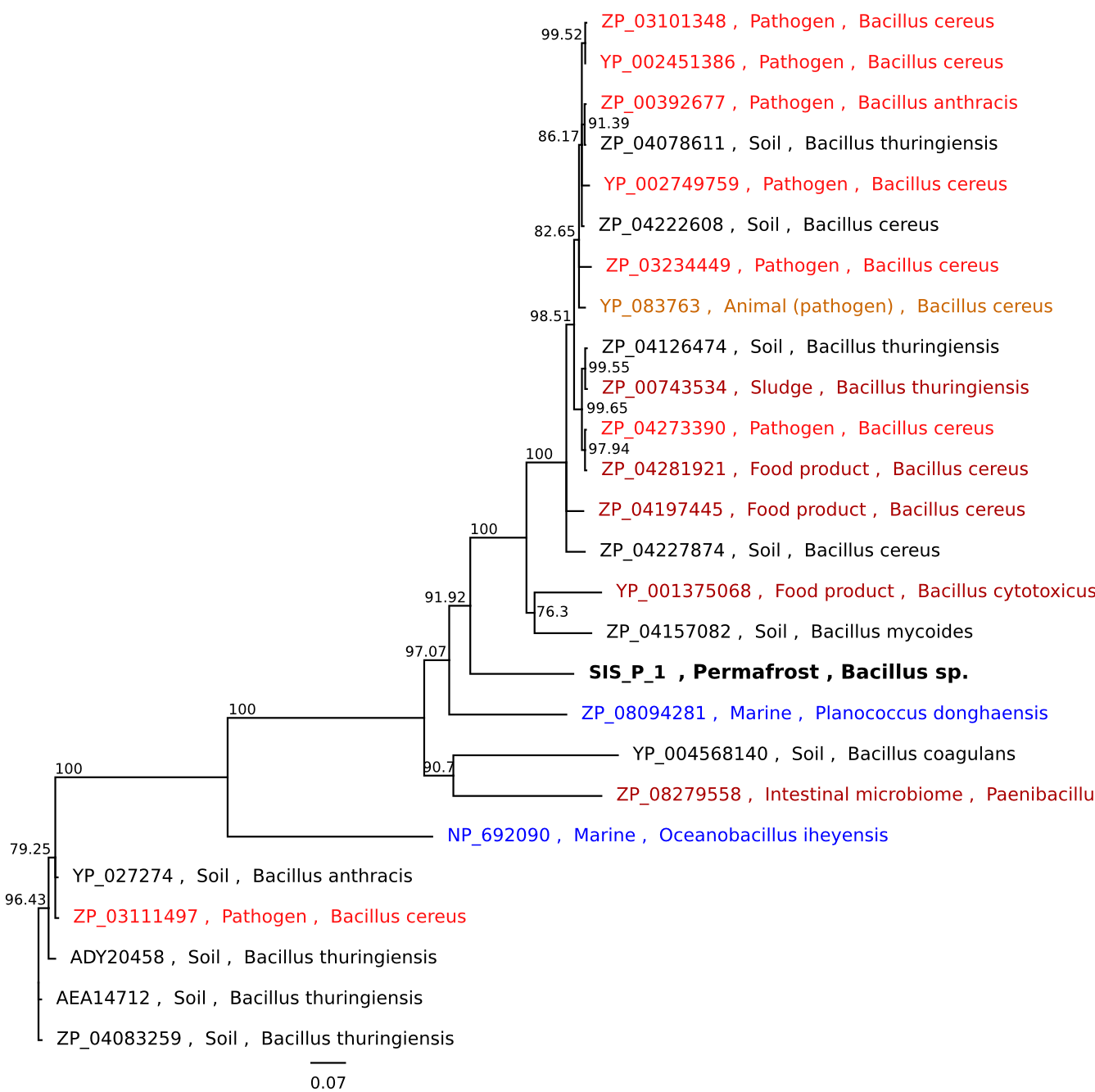


**2)**


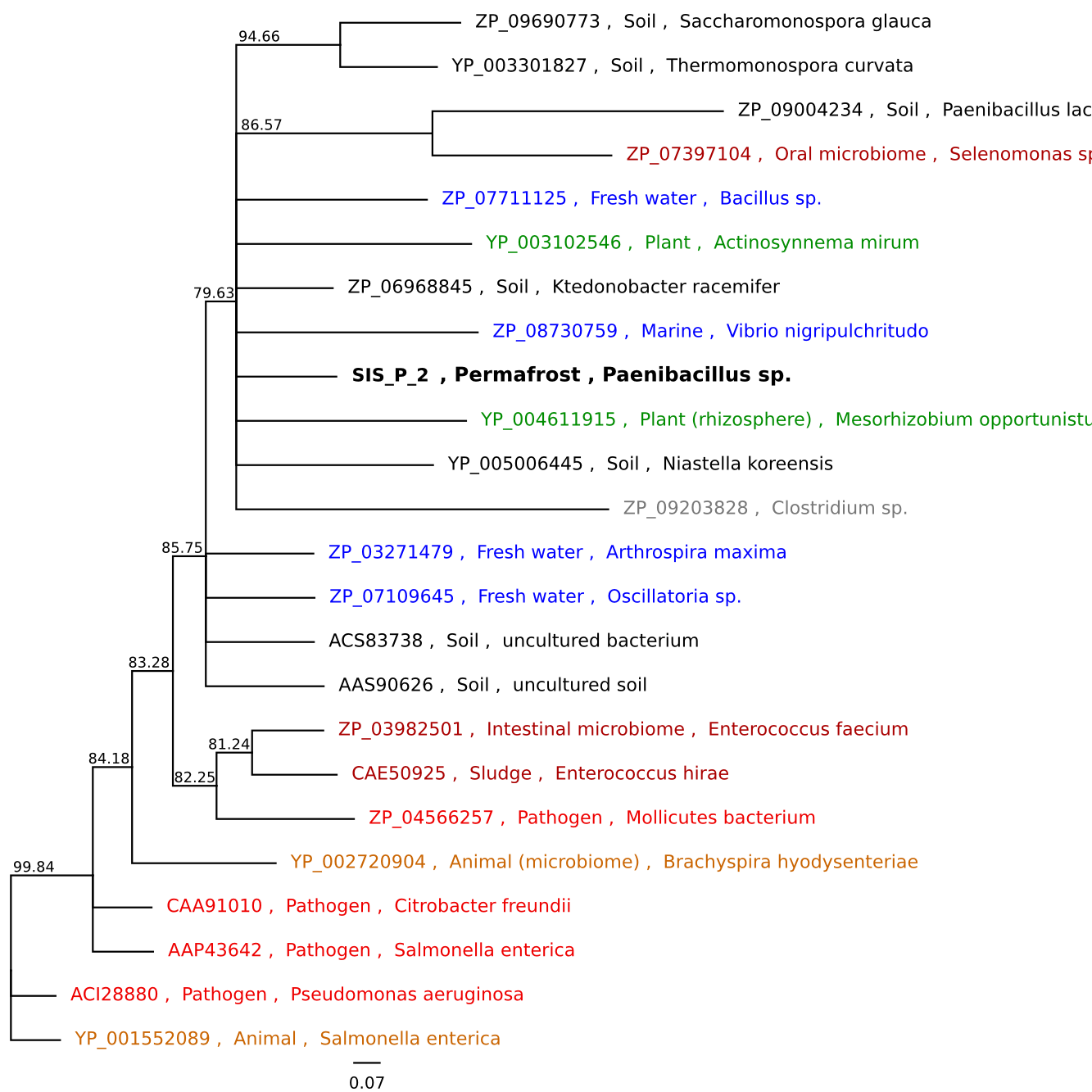


**3)**


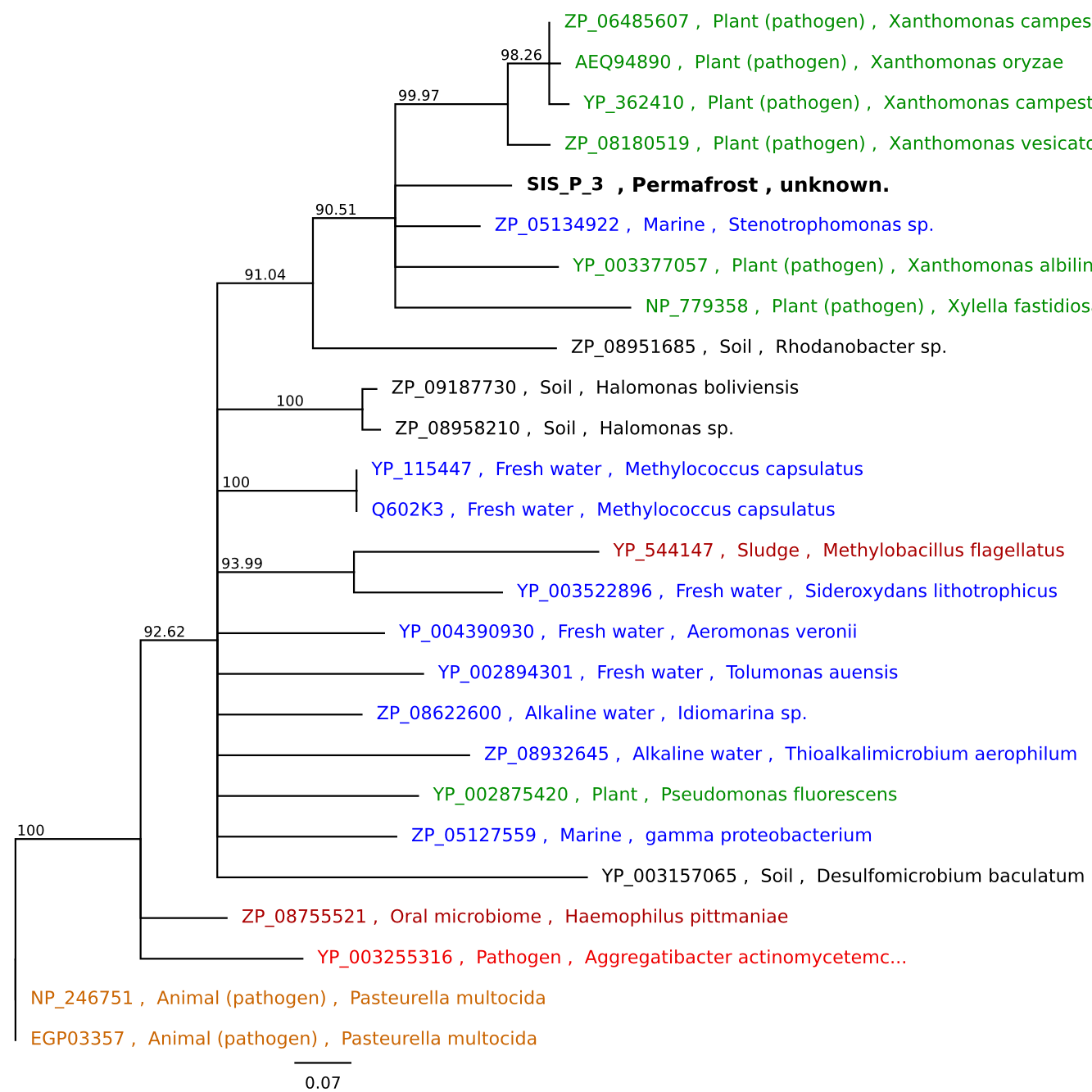


**4)**


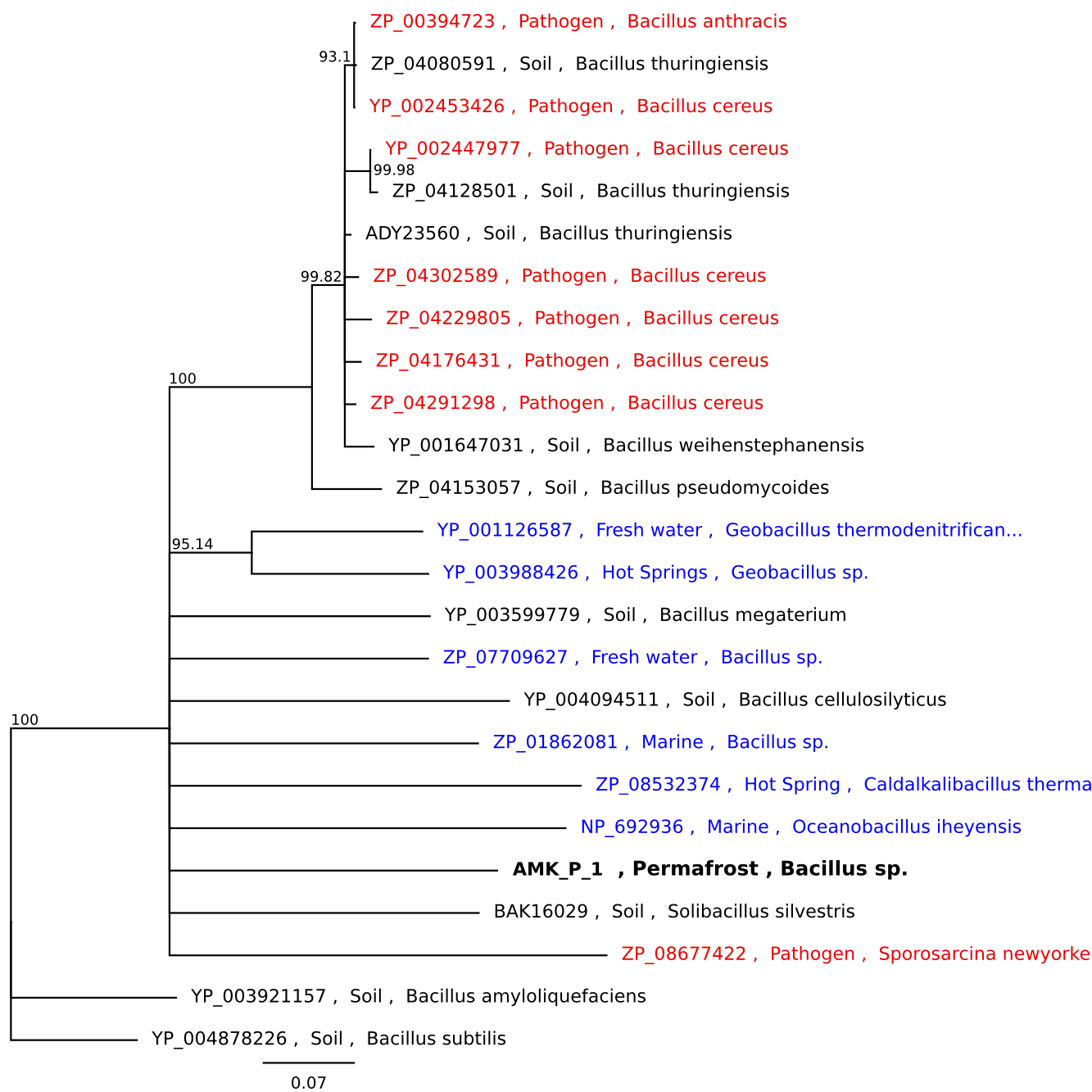


**5)**


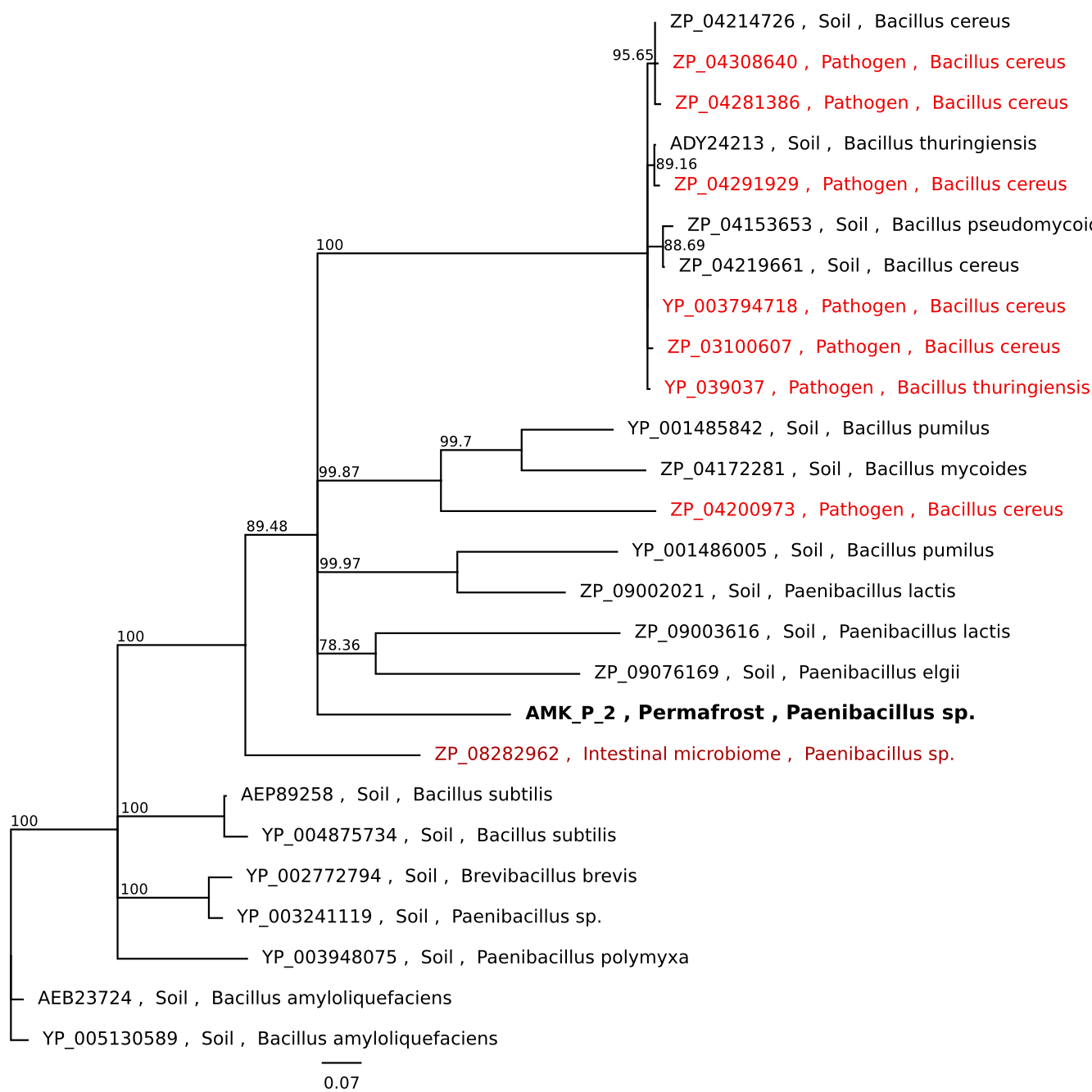


**Figure I**. **Phylogenetic distribution of full-length gene products encoding resistance to aminoglycoside isolated from the Canadian high Arctic active layer soil**: **1)** SIS_AL_1; **2)** SIS_AL_2; **3)** AMK_AL_1; and **4)** AMK_AL_2. To give an overview of the related genes in Genbank, every fourth sequence of the top 100 hits using tblastx were included in the phylogeny. The tip of the branches are identified with the sequence accession number in Genbank, the species of the organism, and when available the source (color code: red is pathogen; dark red is human activity; brown is animal; green is plant; blue is aquatic; and black is soil). Unrooted phylogenetic trees were generated from ClustalW alignment, and a consensus tree (70% of 10,000 bootsraps) was constructed using the neighbor-joining algorithm. Tree branch lengths are proportional to relative sequence identity, and the scale bar is in fixed amino acid substitution per sequence position.

**1)**


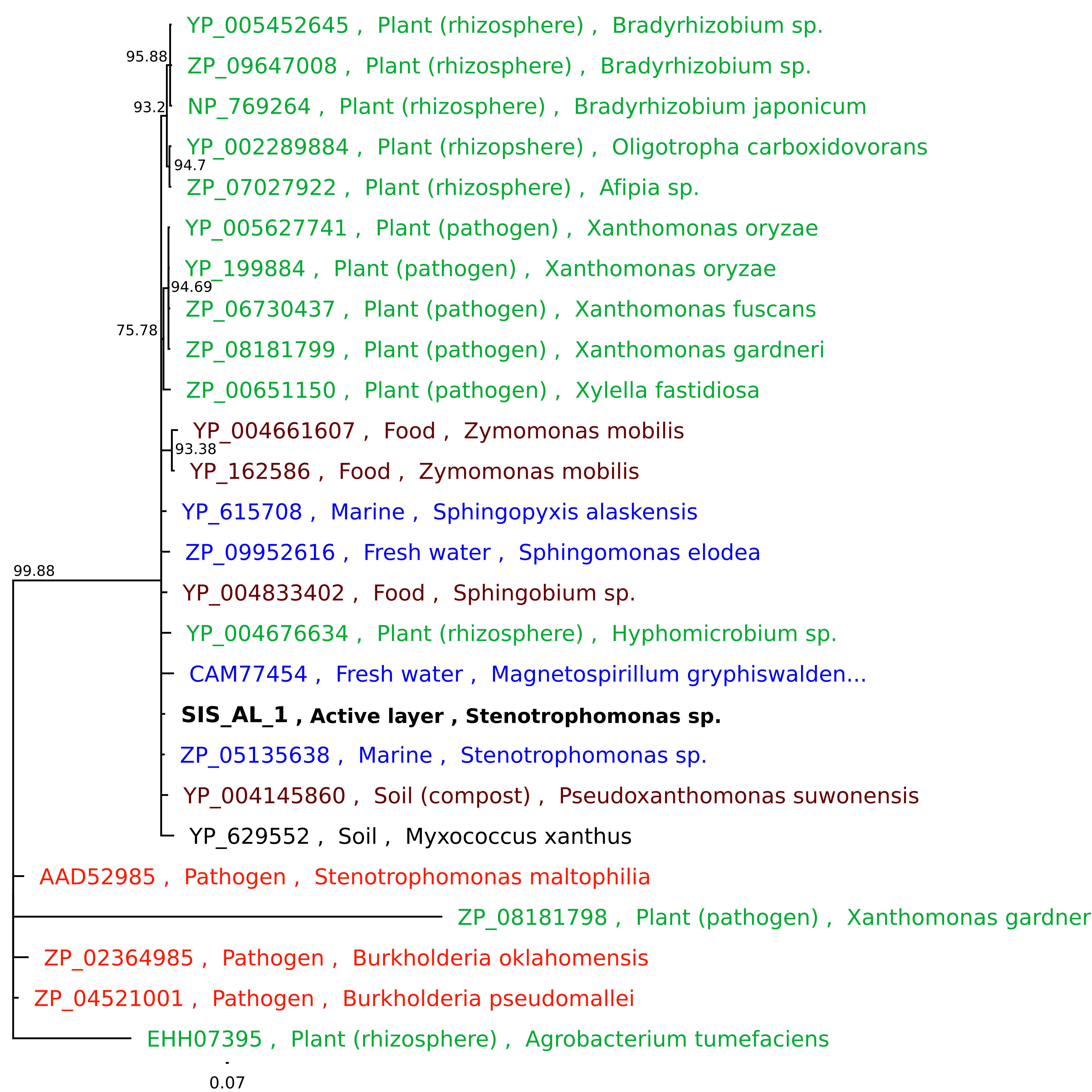


**2)**


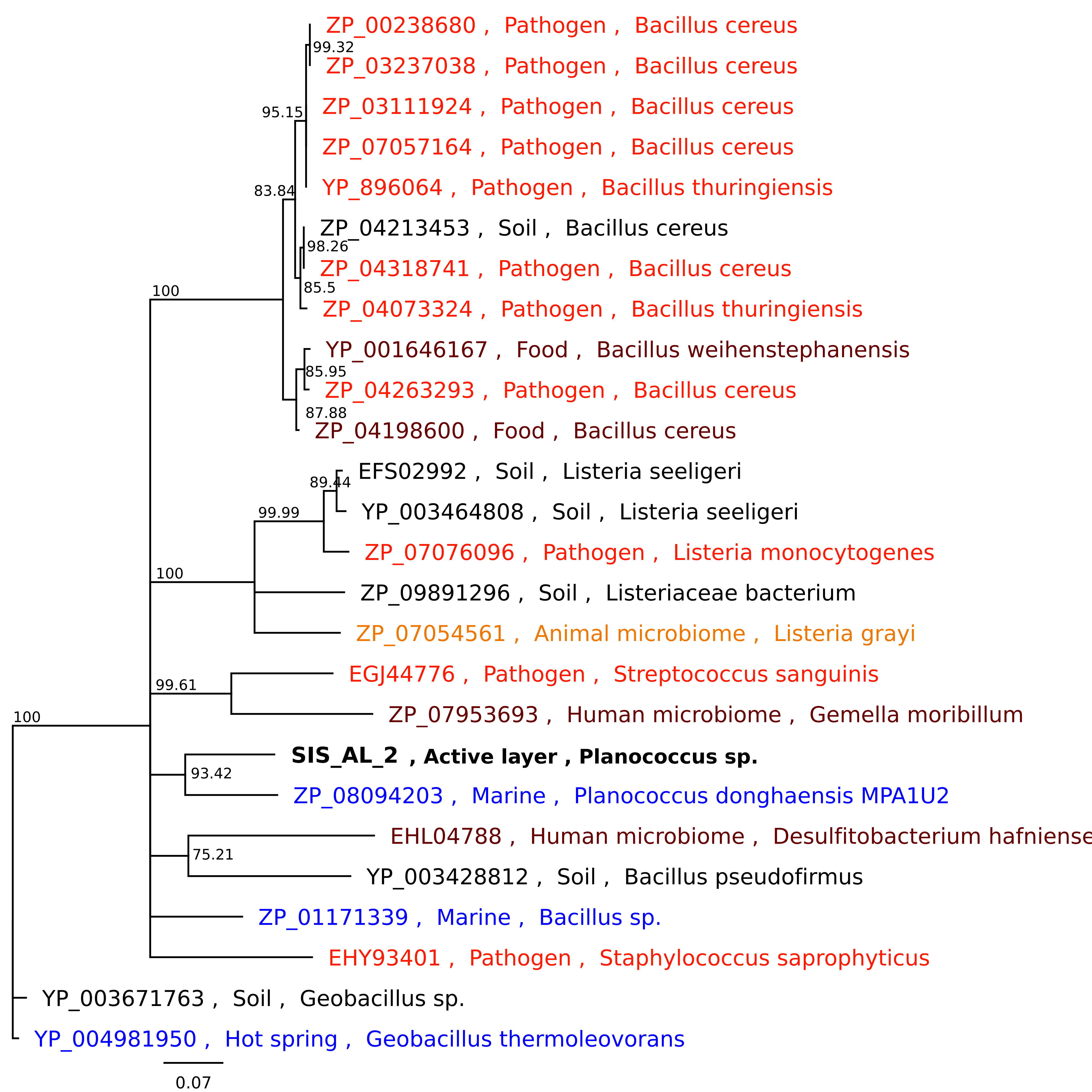


**3)**

**
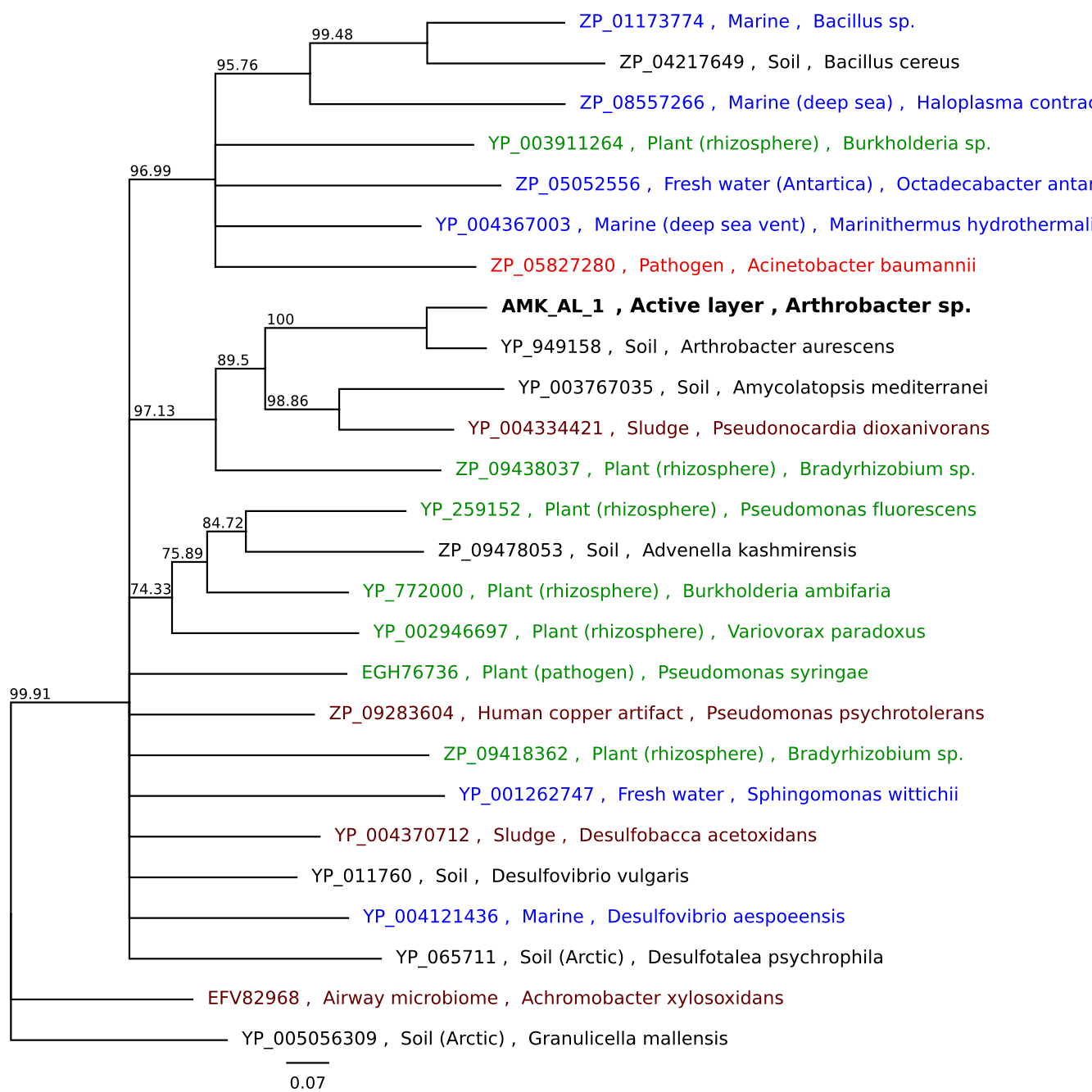
**

**4)**


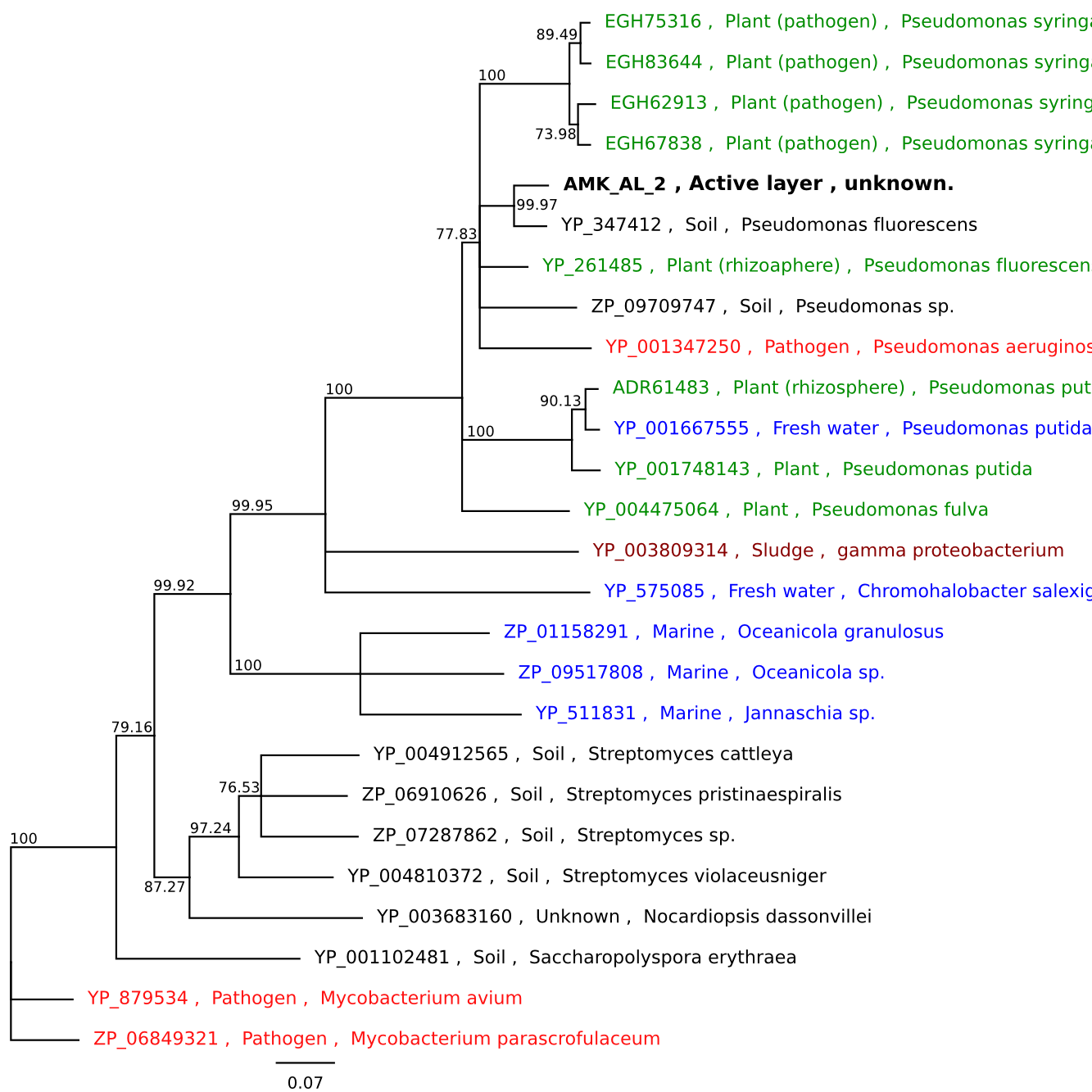


**Figure J. Abundance of putative resistance genes and related proteins at the sampling sites and other metagenomes.** Amino acid sequences homologous to the antibiotic resistance genes found in **1)** ancient permafrost and **2)** its overlaying active layer were used to calculate and compare the relative abundance of each gene in 28 metagenomes. Abundance was calculated as hits per megabase (Mb) and revealed that most resistance genes were found in other environmental metagenomes, but were found to be rare in gut metagenomes. Metagenomes used in this study are described in Table S10: three soil and three marine metagenomes were used while 20 gut metagenomes were used.

**1)**

**
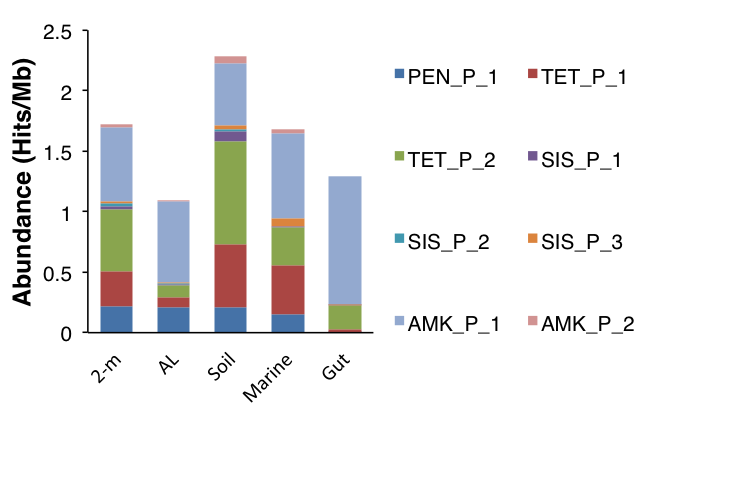
**


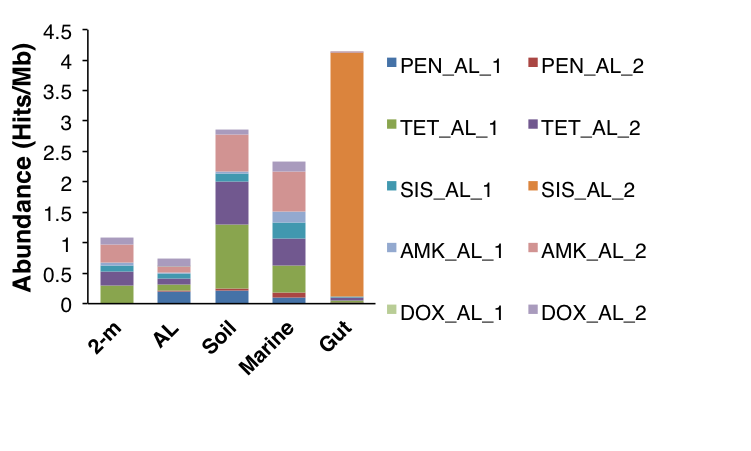
**2)**

**Table A. List of strains, plasmids and primers used for library construction.**

| **Strains** | **Genotype** | **Source** |
| --- | --- | --- |
| *Escherichia coli* | MegaX DH10B | Invitrogen |
| *Escherichia coli* | Low cloning efficiency | Invitrogen |
| *Staphylococcus aureus* | MCD01 | Unpublished lab collection |
| *Staphylococcus epidermidis* | MCD02 | Unpublished lab collection |
|  |  |  |
| **Plasmid** | **Reference** | **Source** |
| pZE21 MCS1 | Sommer *et al*. (2009) | Gautam Dantas |
|  |  |  |
| **Primers** | **Name** | **Sequence** |
| 16S rDNA | 8F | 5′-AGAGTTTGATCCTGGCTCAG-3’ |
|  | 1492R | 5′-TACCTTGTTACGACTT–3’ |
| Sequencing insert | pZE21_81_104 | 5’-GAATTCATTAAAGAGGAGAAAGGT‐3’ |
|  | pZE21_151_174rc | 5’-TTTCGTTTTATTTGATGCCTCTAG-3’ |
| Linearizing primer | pZE21_126_146FOR | 5′-GACGGTATCGATAAGCTTGAT-3′ |
|  | pZE21_111_123rcREV | 5′-GACCTCGAGGGGGGG-3′ |
|  |  |  |

**Table B. Primers used to identify the permafrost bacteria strain(s) harboring each resistant inserts.**

| **Gene** | **Primers** | **Size (bp)** |
| --- | --- | --- |
| PEN_P_1 | 5’-CGCTCTGCGCAGCAGTTCATCT-3’ | 135 |
|  | 5’-CAGGCAGCGCCCCTCGTTTTTA-3’ |  |
| TET_P_1 | 5’-CAGCAAGGGCAGGGCCAATCAT-3’ | 306 |
|  | 5’-CGGGTGGTTTTGTCGGCGTGAT-3’ |  |
| TET_P_2 | 5’-GGTTTAGCAGTGCAGGTAGCGGT-3’ | 496 |
|  | 5’-CCTGCCGTTGTCACAGGCATCA-3’ |  |
| SIS_P_1 | 5’-ATTCCAGACGAGCAGCTTCC-3’ | 371 |
|  | 5’-GCCAACACCACATTTGGCAT-3’ |  |
| SIS_P_2 | 5’-GTTCGCATCCGGCAATACAC-3’ | 364 |
|  | 5’-GGAGGATCGAATCAGCAGGG-3’ |  |
| SIS_P_3 | 5’-CGGGCTGCAGGAATTCGATA-3’ | 332 |
|  | 5’-AGAGGAGAAAGGTACCGGGC-3’ |  |
| AMK_P_1 | 5’-TTGCAAAGGCGGTTCAACTG-3’ | 324 |
|  | 5’-AAAACGAACCGAATTGCGCA-3’ |  |
| AMK_P_2 | 5’-ACCGCAGCGATCGGAAGGAAGA-3’ | 105 |
|  | 5’-ACGTGTTGGAGGCTACGGCTCT-3’ |  |

**Table C. Primers used to identify the active layer bacteria strain(s) harboring each resistant inserts.**

| **Gene** | **Primers** | **Size (bp)** |
| --- | --- | --- |
| PEN_AL_1 | 5’-TTGTCTGGACGATCGGCCTCGT -3’ | 235 |
|  | 5’-AACCGGTCAGCCAAGACACCCT-3’ |  |
| PEN_AL_2 | 5’-GCAAGGCCGACGATTTTGCTGC-3’ | 258 |
|  | 5’-TGCCGATATGCCGCATGGTCAC-3’ |  |
| TET_AL_1 | 5’-GCTGGCGCTGGGTGTTCTTCAT-3’ | 329 |
|  | 5’-CAGCAGCGAGGACAGGCCAAAA-3’ |  |
| TET_AL_2 | 5’-TACATCGCCGGCTCGCCTTTTG-3’ | 353 |
|  | 5’-CAACCAAGCAATGCCGACGCAC-3’ |  |
| SIS_AL_1 | 5’-AGTGAACTGGCCTCGGACAGCA-3’ | 187 |
|  | 5’-TGCATCCAGCACAAGGAGCAGC-3’ |  |
| SIS_AL_2 | 5’-TTCCTACGGTACATGGGCGCGA-3’ | 166 |
|  | 5’-ACTGGATTGTGCCGCTTCCAGC-3’ |  |
| AMK_AL_1 | 5’-TCCATCCGGGCTGGGTGACAAT-3’ | 113 |
|  | 5’-ATTCGATCAGTTCGCCGCTGCC-3’ |  |
| AMK_AL_2 | 5’-GGCGTATTGCGAAGCACTGGGT-3’ | 185 |
|  | 5’-CACGACCGCCCATCAAACCGAT-3’ |  |
| DOX_AL_1 | 5’-CAGACGCTGGACACCTCGTCCC-3’ | 182 |
|  | 5’-GACAGCCAGGGCTCGACCAG-3’ |  |
| DOX_AL_2 | 5’-GATCGAACTGGGCGGCAACGAT-3’ | 183 |
|  | 5’-AGATCCGCATAGGTCTGGCGCA-3’ |  |

**Table D. List of bacteria strains isolated from the permafrost and associated resistance genes.**

| **Strain I.D.** | **Genus** | **16S rRNA**  **Accession #** | **Resistance Gene** |
| --- | --- | --- | --- |
| **Eur3 2.2** | *Sporosarcina sp.* | EU218845 |  |
| **Eur3 2.5** | *Paenibacillus sp.* | EU218847 | SIS_P_2 (); AMK_P_2 () |
| **Eur3 2.6** | *Bacillus sp.* | EU218848 |  |
| **Eur3 2.7** | *Bacillus sp.* |  | TET_P_1 () |
| **Eur3 2.8** | *Bacillus sp.* | EU218849 |  |
| **Eur3 2.10** | *Bacillus sp.* | EU218842 | TET_P_1 () |
| **Eur3 2.11** | *Bacillus sp.* |  | SIS_P_1 (); AMK_P_1 () |
| **Eur3 2.12** | *Staphylococcus sp.* | EU218843 | PEN_P_1 () |
| **Eur3 2.13** | *Paenibacillus* |  |  |
| Eur3 2m Or3 | Unknown |  |  |
| **Eur3 3.29** | *Bacillus sp.* |  | SIS_P_1 (); AMK_P_1 () |
| **Eur3 4m Cl2** | *Paraliobacillus sp.* |  | TET_P_2 () |
| Eur3 9m Wh3 | Unknown |  |  |
| Eur3 14.54.19 | Unknown |  |  |
| **Eur3 14.54.2** | *Paraliobacillus sp.* |  | TET_P_2 () |
| **Eur3 14.54.14** | *Bacillus sp.* |  | TET_P_1 () |
| **Eur3 14.54.6** | *Sporosarcina sp.* |  |  |
| **Eur3 14.54.7** | *Bacillus sp.* |  |  |

* Strain ID refers to a unique identifier in Stevens et al. (2008).

**Table E. List of bacteria strains isolated from active layer and associated resistance genes.**

| **Strain I.D.** | **Genus** | **Accession #** | **Resistance Gene** |
| --- | --- | --- | --- |
| Eur3 AL.1 | *Actinobacterium* sp. | EU218851 |  |
| Eur3 AL.2 | *Flavobacterium* sp. | EU218855 |  |
| Eur3 AL.3 | *Arthrobacter* sp. |  | TET_AL_1 (); SIS_AL_2 () |
| Eur3 AL.5 | *Stenotrophomonas* sp. | EU218838 | PEN_AL_1 (); PEN_AL 2 (); SIS_AL_1 (); DOX_AL_1 (); DOX_AL_2 () |
| Eur3 AL.6 | *Stenotrophomonas* sp. |  | PEN_AL_2 (); SIS_AL_1 (); DOX_AL1 (); DOX_AL_2 () |
| Eur3 AL.7 | *Arthrobacter* sp. |  |  |
| Eur3 AL.9 | *Arthrobacter* sp. |  |  |
| Eur3 AL.10 | *Arthrobacter* sp. | EU218852 |  |
| Eur3 AL.11 | *Methylobacterium* sp. |  |  |
| Eur3 AL.14 | *Planococcus* sp. | EU218853 | SIS_AL_2 () |
| Eur3 AL.16 | *Pseudomonas* sp. | EU218854 |  |
| Eur3 AL.17 | *Arthrobacter* sp. |  |  |
| Eur3 AL.18 | *Arthrobacter* sp. |  |  |
| Eur3 AL.19 | *Pseudomonas* sp. |  | TET_AL_2 () |
| Eur3 AL.24 | *Arthrobacter* sp. | EU218856 |  |
| Eur3 AL.25 | *Arthrobacter* sp. | EU218857 | TET_AL_1 () |
| Eur3 AL.26 | *Rhodoglobus* sp. | EU218858 |  |
| Eur3 AL.28 | *Paenisporosarcina* sp. | EU218860 |  |
| Eur3 AL.30 | *Arthrobacter* sp. | EU218861 |  |
| Eur3 AL.31 | *Pedobacter* sp. | EU218862 |  |
| Eur3 AL.32 | *Flavobacterium* sp. |  |  |
| Eur3 AL.34 | *Arthrobacter* sp. | EU218872 | AMK_AL_1 () |
| Eur3 AL.35 | *Arthrobacter* sp. |  | TET_AL_1 () |
| Eur3 AL.36 | *Salinibacterium* sp. |  |  |
| Eur3 AL.40 | *Salinibacterium* sp. |  |  |

* Strain ID refers to a unique identifier in Stevens et al. (2008).

**Table F. Numbers of antibiotic resistant clones sequenced and unique resistance genes found from a functional analysis of the permafrost and the active layer of the Canadian high Arctic.**

|  | **Permafrost** | | **Active Layer** | |
| --- | --- | --- | --- | --- |
| **Antibiotics** | **Sequenced** | **Unique** | **Sequenced** | **Unique** |
| Amikacin | 5 | 2 | 5 | 2 |
| Carbenicillin | n.a | n.a | 5 | 2 |
| Doxycycline | n.a | n.a | 5 | 2 |
| Penicillin | 5 | 1 | 5 | 2 |
| Sisomicin | 5 | 3 | 5 | 2 |
| Tetracycline | 5 | 2 | 5 | 2 |

**Table G**. **Resistance genes identified using metagenomic functional selections from Canadian High Arctic permafrost.** Gene ID is constituted from a three-letter code for the antibiotics used for the selections (e.g. AMK denotes amikacin, see Table S1), P denotes permafrost and a numeric identifier. For each gene identified, the most similar gene from any organism as well as the most similar gene harbored by a pathogenic isolate in GenBank was identified using tblastx. Global sequence identities at the nucleotide and amino acid level between these genes were computed using clustalW (3).

| **Gene ID**  **(symbol)** | **Gene length [bp]** | **GenBank ID** | **Gene Annotation** | **Top hit**  **[gbID|title|position]** | **Global DNA % ID** | **Global Protein % ID** | **Top pathogenic hit**  **[gbID|title|position]** | **Global DNA % ID** | **Global Protein % ID** |
| --- | --- | --- | --- | --- | --- | --- | --- | --- | --- |
| AMK_P_1 | 1,125 | KC520481 | Aminotransferase class V | CP001638.1|Geobacillus_sp_WcH70|2523741-2524886 | 41.9 | 64.1 | AE017194.1  Bacillus cereus ATCC 10987, 4149349-4150494 | 39.7 | 59.3 |
| AMK_P_2 | 642 | KC520482 | Putative transporter | AP008955.1|Brevibacillus_brevis_NBRC_100599|370375-371541 | 39.9 | 46.4 | CP001177.1  Bacillus cereus AH187, 4762080-4763252 | 42.5 | 37.5 |
| PEN_P_1 | 1,701 | KC520475 | Penicllin acylase II | CP001907.1  Bacillus thuringiensis serovar chinensis CT-43 3219878-3222259 | 63.2 | 60.8 | CP001176.1  Bacillus cereus B4264, 3155458-3157848 | 62.7 | 60.4 |
| SIS_P_1 | 1,362 | KC520478 | Protoporphyrinogen oxidase | CP000903.1|Bacillus_weihenstephanensis_KBAB4|2297537-2298937 | 67.1 | 65.2 | AE017194.1  Bacillus cereus ATCC 10987, 2337571-2338971 | 67.4 | 65.2 |
| SIS_P_2 | 591 | KC520479 | aminoglycoside N(6')-acetyltransferase (AAC(6')), putative | CP000828.1|  Acaryochloris_marina_MBIC11017| 4380776-4381336 | 56.5 | 51.6 | L12710.1| Enterococcus faecium|1-1485 | 49.2 | 40.1 |
| SIS_P_3 | 345 | KC520480 | Porphobilinogen deaminase | CP001111.1|Stenotrophomonas maltophilia R551-3|3968310--3969221 | 47.6 | 51.7 | AM743169.1 |Stenotrophomonas maltophilia K279a|423884-4239759 | 47.0 | 49.3 |
| TET_P_1 | 1,188 | KC520476 | permease of the major facilitator superfamily | AP012157.1|  Solibacillus silvestris|  3233596-3234783 | 62.4 | 61.3 | CP000001.1|Bacillus_cereus| 829799-831001 | 55.0 | 43.4 |
| TET_P_2 | 1,431 | KC520477 | putative drug antiporter (transporter) | CP000002.3  Bacillus licheniformis 3369265-33707 | 60.6 | 59.0 | AP008934.1  Staphylococcus saprophyticus subsp. saprophyticus 2457800-2459203 | 59.8 | 52.4 |

**Table H**. **Resistance genes identified using metagenomic functional selections from Canadian High Arctic active layer.** Gene ID is constituted from a three-letter code for the antibiotics used for the selections (e.g. AMK denotes amikacin, see Table S1), AL denotes active layer, and a numeric identifier. For each gene identified, the most similar gene from any organism as well as the most similar gene harbored by a pathogenic isolate in GenBank was identified using tblastx. Global sequence identities at the nucleotide and amino acid level between these genes were computed using clustalW (3).

| **Gene ID** | **Gene length [bp]** | **GenBank ID** | **Gene Annotation** | **Top hit**  **[gbID|title|position]** | **Global DNA % ID** | **Global Protein % ID** | **Top pathogenic hit**  **[gbID|title|position]** | **Global DNA % ID** | **Global Protein % ID** |
| --- | --- | --- | --- | --- | --- | --- | --- | --- | --- |
| AMK_AL_1 | 624 | KC520489 | Putative acyl carrier protein phosphodiesterase | CP000474.1|Arthrobacter_  aurescens_TC1|  3816658-3817335 | 74.7 | 79.7 | AM747722.1  Burkholderia cenocepacia J2315 201784-202413 | 47.7 | 33.0 |
| AMK_AL_2 | 963 | KC520490 | Putative dehydrogenase | CP000094.2|Pseudomonas_  fluorescens_Pf01-|  1876884-1877846 | 85.3 | 91.9 | CP000744.1  Pseudomonas aeruginosa PA7, 1908293-1909255 | 72.7 | 77.3 |
| DOX_AL_1 | 475 | KC520491 | Glycerol-3-phosphate O-acyltransferase | CP001111.1  Stenotrophomonas maltophilia R551-3, 64096-66732 | 90.7 | 94.3 | AM743169.1  Stenotrophomonas maltophilia K279a 99901..102528 | 72.5 | 68.3 |
| DOX_AL_2 | 573 | KC520492 | Acyl-CoA thioesterase I | AM743169.1  Stenotrophomonas maltophilia K279a 843424-844068 | 89.7 | 94.3 | AM743169.1  Stenotrophomonas maltophilia K279a 843424-844068 | 89.7 | 94.3 |
| PEN_AL_1  CAR_AL_1 | 1,167 | KC520483 | Putative beta-lactamase family protein | CP000744.1  Pseudomonas aeruginosa PA7, 987337-988530 | 78.7 | 73.7 | CP000744.1  Pseudomonas aeruginosa PA7, 987337-988530 | 78.7 | 73.7 |
| PEN_AL_2  CAR_AL_2 | 957 | KC520484 | L2 beta-lactamase | EU032534|Stenotrophomonas_maltophilia_HK|1042-1953 | 72.5 | 68.3 | EU032534|Stenotrophomonas_maltophilia_HK|1042-1953 | 72.5. | 68.3 |
| SIS_AL_1 | 1,130 | KC520487 | Aminoglycoside 6’-N-acetyltransferase lz | AF140221.1|Stenotrophomas maltophilia  1-1104 | 71.1 | 52.3 | AF140221.1|Stenotrophomas maltophilia  1-1104 | 71.1 | 52.3 |
| SIS_AL_2 | 561 | KC520488 | Multidrug ABC transporter ATPase and permease | NZ_AEPB01000022.1|Planococcus donghaensis MPA1U2|61374-63128 | 48.5 | 81.1 | NZ_AARP04000033.1|Listeria monocytogenes FSL N1-017|6501-7367 | 58.2 | 64.7 |
| TET_AL_1 | 1,162 | KC520485 | drug resistance transporter, EmrB/QacA | CP002379.1|Arthrobacter_phenanthrenivorans_Sphe3|4075000-4076652 | 85.3 | 92.2 | CP001802.1  Gordonia bronchialis DSM 43247, 4322485-4324173 | 62.7 | 69.9 |
| TET_AL_2 | 1,197 | KC520486 | Putative transporter | CP002585.1  Pseudomonas brassicacearum subsp. brassicacearum NFM421, 820308-821510 | 79.8 | 86.5 | FM209186.1  Pseudomonas aeruginosa LESB58 1575805-1576983 | 44.7 | 47.9 |

**Table I. List of environmental microbiomes used for studying the distribution of each resistant insert.**

| **Project ID** | **MG-RAST ID** | **Source** | **Identified protein features** | **Reference** |
| --- | --- | --- | --- | --- |
| 2m | 4443232.3 | Eur3 2m permafrost, Canada | 198,064 | Stevens *et al*. 2008 |
| AL | 4443231.3 | Eur3 Active layer, Canada | 870,505 | Stevens *et al*. 2008 |
| Soil1 | 4441091.3 | Soil, Farm, MN, USA | 107,795 | Tringe *et al*. 2005 |
| Soil2 | 4450750.3 | Soil, NV, USA | 190,050 | - |
| Soil3 | 4446153.3 | Soil, rain forest, PR, USA | 677,007 | PI: Terry Hazen |
| Marine1 | 4441579.3 | Marine, Gulf of Maine, Canada | 170,301 | Rusch *et al*. 2010 |
| Marine2 | 4441594.3 | Marine, Galapagos, Ecuador | 146,860 | Rusch *et al*. 2010 |
| Marine3 | 4441571.3 | Marine, Saragossa Sea, Bermuda | 881,000 | Rusch *et al*. 2010 |
| TS1 | 4440452.7 | Human gut microbiome | 181,643 | Turnbaugh *et al*. 2009 |
| TS2 | 4440453.6 | Human gut microbiome | 319,071 | Turnbaugh *et al*. 2009 |
| TS3 | 4440595.4 | Human gut microbiome | 376,310 | Turnbaugh *et al*. 2009 |
| TS4 | 4440460.5 | Human gut microbiome | 285,992 | Turnbaugh *et al*. 2009 |
| TS5 | 4440461.5 | Human gut microbiome | 328,464 | Turnbaugh *et al*. 2009 |
| TS6 | 4440462.5 | Human gut microbiome | 385,935 | Turnbaugh *et al*. 2009 |
| TS7 | 4440823.3 | Human gut microbiome | 307,201 | Turnbaugh *et al*. 2009 |
| TS8 | 4440824.3 | Human gut microbiome | 338,816 | Turnbaugh *et al*. 2009 |
| TS9 | 4440826.3 | Human gut microbiome | 382,791 | Turnbaugh *et al*. 2009 |
| TS19 | 4440610.3 | Human gut microbiome | 306,687 | Turnbaugh *et al*. 2009 |
| TS20 | 4440611.3 | Human gut microbiome | 334,505 | Turnbaugh *et al*. 2009 |
| TS21 | 4440639.3 | Human gut microbiome | 312,690 | Turnbaugh *et al*. 2009 |
| TS28 | 4440613.3 | Human gut microbiome | 219,559 | Turnbaugh *et al*. 2009 |
| TS29 | 4440616.3 | Human gut microbiome | 347,012 | Turnbaugh *et al*. 2009 |
| TS30 | 4440825.3 | Human gut microbiome | 355,099 | Turnbaugh *et al*. 2009 |
| TS49 | 4440614.3 | Human gut microbiome | 356,059 | Turnbaugh *et al*. 2009 |
| TS50 | 4440615.3 | Human gut microbiome | 344,663 | Turnbaugh *et al*. 2009 |
| TS51 | 4440640.3 | Human gut microbiome | 314,244 | Turnbaugh *et al*. 2009 |
|  |  |  |  |  |

Steven B, Pollard WH, Greer CW, & Whyte LG (2008) Microbial diversity and activity through a permafrost/ground ice core profile from the Canadian high Arctic. *Environ Microbiol* 10(12):3388-3403.

Tringe SG*, et al.* (2005) Comparative metagenomics of microbial communities. *Science* 308(5721):554-557.

Rusch DB, Martiny AC, Dupont CL, Halpern AL, & Venter JC (2010) Characterization of Prochlorococcus clades from iron-depleted oceanic regions. *Proc Natl Acad Sci U S A* 107(37):16184-16189.

Turnbaugh PJ*, et al.* (2009) A core gut microbiome in obese and lean twins. *Nature* 457(7228):480-484.

**Table J. Growth profile of bacterial isolate at different temperatures**

|  |  | ***psychrotrophic*** | |  |  |
| --- | --- | --- | --- | --- | --- |
| **Isolate** | **Genus** | **0°C** | **5°C** | **21°C** | **37°C** |
| Eur3 2.8 | *Bacillus* | + | ++ | ++ | - |
| Eur3 2.12 | *Staphylococcus* | - | + | ++ | + |

**Growth is represented as (+) and absence of growth as (-)**

**Table K. Number of significant BLASTP hits across environmental microbiomes.**

| **Gene** | **2-m** | **AL** | **Soil1** | **Soil2** | **Soil3** | **Marine1** | **Marine2** | **Marine3** |
| --- | --- | --- | --- | --- | --- | --- | --- | --- |
| PEN_P_1 | 9 | 94 | 38 | 13 | 79 | 24 | 22 | 47 |
| TET_P_1 | 12 | 34 | 88 | 55 | 113 | 29 | 26 | 500 |
| TET_P2 | 21 | 43 | 75 | 123 | 214 | 8 | 12 | 500 |
| SIS_P_1 | 1 | 6 | 13 | 10 | 17 | 0 | 1 | 2 |
| SIS_P_2 | 1 | 4 | 3 | 1 | 0 | 0 | 0 | 0 |
| SIS_P_3 | 1 | 1 | 6 | 5 | 2 | 2 | 13 | 57 |
| AMK_P_1 | 25 | 297 | 90 | 45 | 144 | 65 | 98 | 450 |
| AMK_P_2 | 1 | 1 | 3 | 12 | 5 | 0 | 0 | 64 |
| PEN_AL_1 | 0 | 88 | 56 | 13 | 43 | 2 | 7 | 134 |
| PEN_AL_2 | 0 | 4 | 1 | 8 | 6 | 3 | 1 | 160 |
| TET_AL_1 | 12 | 44 | 81 | 168 | 215 | 8 | 11 | 750 |
| TET_AL_2 | 10 | 43 | 90 | 102 | 131 | 13 | 13 | 750 |
| SIS_AL_1 | 4 | 40 | 18 | 17 | 27 | 36 | 34 | 137 |
| SIS_AL_2 | 123 | 700 | 418 | 300 | 601 | 394 | 500 | 850 |
| AMK_AL_1 | 2 | 3 | 2 | 4 | 5 | 21 | 15 | 146 |
| AMK_AL_2 | 12 | 49 | 69 | 73 | 171 | 76 | 58 | 560 |
| DOX_AL_1 | 0 | 1 | 0 | 0 | 0 | 0 | 0 | 0 |
| DOX_AL_2 | 5 | 57 | 23 | 4 | 16 | 22 | 11 | 128 |
|  |  |  |  |  |  |  |  |  |

**Table L. Number of significant BLASTP hits across gut microbiomes.**

| **Gene** | **TS1** | **TS2** | **TS3** | **TS4** | **TS5** | **TS6** | **TS7** | **TS8** | **TS9** | **TS19** | **TS20** | **TS21** | **TS28** | **TS29** | **TS30** | **TS49** | **TS50** | **TS51** |
| --- | --- | --- | --- | --- | --- | --- | --- | --- | --- | --- | --- | --- | --- | --- | --- | --- | --- | --- |
| PEN_P_1 | 0 | 0 | 0 | 0 | 0 | 0 | 0 | 0 | 0 | 0 | 0 | 0 | 0 | 0 | 0 | 0 | 1 | 0 |
| TET_P_1 | 1 | 1 | 0 | 4 | 0 | 2 | 2 | 0 | 3 | 2 | 1 | 1 | 4 | 18 | 4 | 1 | 4 | 6 |
| TET_P2 | 10 | 15 | 10 | 14 | 6 | 13 | 9 | 14 | 5 | 2 | 7 | 9 | 24 | 101 | 8 | 34 | 83 | 49 |
| SIS_P_1 | 0 | 0 | 0 | 0 | 0 | 0 | 0 | 1 | 0 | 0 | 2 | 0 | 0 | 0 | 0 | 0 | 0 | 0 |
| SIS_P_2 | 0 | 0 | 0 | 0 | 0 | 0 | 0 | 0 | 0 | 0 | 0 | 0 | 0 | 0 | 0 | 0 | 0 | 0 |
| SIS_P_3 | 0 | 0 | 1 | 1 | 3 | 0 | 1 | 1 | 0 | 0 | 0 | 0 | 3 | 2 | 0 | 1 | 1 | 0 |
| AMK_P_1 | 85 | 85 | 132 | 48 | 141 | 122 | 177 | 161 | 149 | 29 | 99 | 89 | 143 | 203 | 81 | 117 | 125 | 69 |
| AMK_P_2 | 0 | 0 | 0 | 0 | 0 | 0 | 0 | 0 | 0 | 0 | 0 | 0 | 0 | 0 | 0 | 0 | 0 | 0 |
| PEN_AL_1 | 0 | 0 | 4 | 1 | 2 | 1 | 0 | 0 | 1 | 0 | 0 | 1 | 2 | 1 | 0 | 0 | 0 | 0 |
| PEN_AL_2 | 1 | 1 | 0 | 0 | 3 | 1 | 0 | 1 | 0 | 0 | 0 | 1 | 0 | 1 | 1 | 2 | 0 | 0 |
| TET_AL_1 | 1 | 3 | 7 | 2 | 2 | 2 | 4 | 5 | 1 | 1 | 1 | 1 | 8 | 27 | 0 | 4 | 2 | 3 |
| TET_AL_2 | 4 | 1 | 9 | 3 | 1 | 8 | 2 | 8 | 1 | 1 | 4 | 4 | 7 | 20 | 4 | 3 | 10 | 6 |
| SIS_AL_1 | 2 | 1 | 4 | 5 | 1 | 4 | 1 | 3 | 2 | 4 | 0 | 0 | 1 | 1 | 6 | 0 | 1 | 7 |
| SIS_AL_2 | 341 | 301 | 413 | 175 | 456 | 405 | 604 | 599 | 604 | 172 | 367 | 313 | 603 | 506 | 407 | 412 | 601 | 354 |
| AMK_AL_1 | 0 | 0 | 1 | 0 | 0 | 0 | 0 | 0 | 0 | 0 | 0 | 0 | 0 | 0 | 0 | 0 | 0 | 0 |
| AMK_AL_2 | 0 | 2 | 0 | 0 | 0 | 2 | 0 | 2 | 1 | 0 | 0 | 0 | 2 | 6 | 1 | 0 | 1 | 0 |
| DOX_AL_1 | 0 | 0 | 0 | 0 | 0 | 0 | 0 | 0 | 0 | 0 | 0 | 0 | 0 | 0 | 0 | 0 | 0 | 0 |
| DOX_AL_2 | 0 | 0 | 0 | 0 | 0 | 0 | 0 | 0 | 0 | 0 | 0 | 0 | 0 | 0 | 0 | 0 | 0 | 0 |
|  |  |  |  |  |  |  |  |  |  |  |  |  |  |  |  |  |  |  |
